# Tailoring Waterborne Coating Rheology with Hydrophobically Modified Ethoxylated Urethanes (HEURs): Molecular Architecture Insights Supported by CG-MD Simulations

**DOI:** 10.1021/acs.iecr.4c00253

**Published:** 2024-05-24

**Authors:** Ioanna Tzortzi, Imane Joundi, Michail Kavousanakis, Theodora Spyriouni, Ariana Bampouli, Guillaume Michaud, Tom Van Gerven, Georgios D. Stefanidis

**Affiliations:** † School of Chemical Engineering National Technical University of Athens, Iroon Polytecneiou 9, Zografou Campus, Athens 157 80, Greece; ‡ Department of Chemical Engineering, KU Leuven, Celestijnenlaan 200F, Leuven B-3001, Belgium; § COATEX SAS, 69730 Genay, France; ∥ SCIENOMICS SAS, 16 Rue de l’Arcade, Paris 75008, France

## Abstract

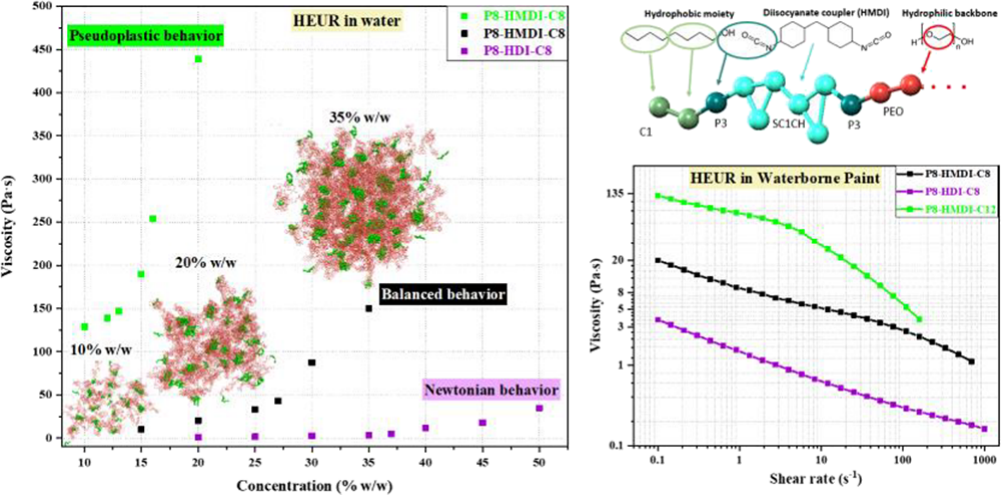

A novel investigation of the effects of the hydrophilic and hydrophobic segments of hydrophobically modified ethoxylated urethanes (HEURs) on the rheological properties of their aqueous solutions, latex-based emulsions, and waterborne paints is demonstrated. Different HEUR thickeners were produced by varying the poly(ethylene glycol) (PEG) molecular weight and terminal hydrophobic size. Results reveal that the strength of hydrophobic associations and, consequently, the rheological properties of HEUR formulations can be effectively controlled by modifying the structure of the hydrophobic segment, specifically, the combination of diisocyanate and monoalcohol. This allows for the on-demand attainment of diverse rheological behaviors ranging from predominantly Newtonian profiles exhibiting lower viscosities to markedly pseudoplastic behaviors with significantly higher viscosities. The length of the hydrophilic group appears to affect viscosity only marginally up to a molecular weight of 23,000 g/mol, with more notable effects at 33,000 g/mol. Additionally, it was indicated that the rheological responses observed in water solutions provide a reliable forecast of their behavior in latex-based emulsions and waterborne paints. Coarse-grained molecular dynamics (CG-MD) simulations were also applied to gain insight into HEUR micelle dynamics in aqueous solutions. Guided by the DBSCAN algorithm, the simulations successfully captured the concentration-dependent behavior and the impact of hydrophilic chain length, aligning with the experimental viscosity trends. Various metrics were employed to provide a comprehensive analysis of the micellization process, including the hydrophobic cluster volume, the total micellar volume, the aggregation number, and the number of chains interconnecting with other micelles.

## 1 Introduction

Waterborne coatings, prevalent in both commercial and residential sectors, owe their unique characteristics to their complex formulation that contain various components like thickeners, latex, dispersing agents, pigments, surfactants, and other ingredients.^1−3^ The rheological behavior of these paints is dictated by the synergistic interactions among these ingredients. Therefore, the optimization of the ingredient selection at minimum cost is challenging, and understanding of the role of raw materials used and their interactions is essential, as compositional and chemical variations can significantly affect the end product.^4^

In this context, rheology modifiers (or viscosity thickeners) exert significant influence on the rheological properties of waterborne dispersions despite their relatively low concentration in the paint formulation (1 to 3 wt %).^1−3,5−7^ Among various rheology modifiers, hydrophobically modified ethoxylated urethanes (HEURs) constitute a specific class of nonionic associative thickeners. They are extensively employed in waterborne paints, inks, emulsions, and coatings due to their superior performance attributes, including excellent flow, leveling, spatter and water resistance, and pH insensitivity. Owing to their amphiphilic polymer structure, HEURs facilitate the formation of dynamic transient networks with hydrophobic components like surfactants, latex, and pigments,^8−15^ whereas the structural composition of the employed HEUR can lead to either Newtonian or pseudoplastic rheological behavior in the final product.^16−18^

Research on the rheological behavior of HEURs in waterborne systems has mostly focused on investigating the rheology of HEURs in aqueous media, both in the absence and in the presence of surfactants.^19−29^ These studies aim to assess the effect of various factors, such as the size of hydrophilic and hydrophobic segments, temperature, concentration, and molecular weight distribution of HEUR and the interaction with surfactants. Further, relevant research activity has been extended to examine the rheology and stability of latex emulsions thickened with HEURs, focusing on the effect of latex monomer composition, particle size distribution, surface charge, and hydrophobicity.^6,13,30−37^ Bridging mechanisms and associations between HEURs and latex particles have also been studied, particularly focusing on the adsorption of hydrophobic segments, interparticle bridging, and loop formation.^12,38,39^ Despite this extensive research, only few studies have transitioned these findings to waterborne paint formulations;^2,3,9,40^ therefore, a comprehensive analysis linking the HEUR rheological behavior across aqueous solutions, latex-based emulsions, and paint formulations is missing. At the same time, the methods for optimizing rheological properties in waterborne dispersions in the industry rely almost exclusively on trial-and-error processes and the experience of formulators, underscoring the necessity for more standardized approaches.

To address the existing knowledge gap in both the scientific and industrial domain, our research analyzes the effects of hydrophilic and hydrophobic segments of HEURs across various waterborne dispersions, including aqueous solutions, latex-based emulsions, and commercial waterborne paint formulations. The primary objective is to establish a structure–rheology relationship for HEURs, facilitating the transition from empirical methods to evidence-based optimization of the rheological performance in waterborne dispersions. The approach to that end is threefold. First, we study how different HEUR structures affect the viscosity profile of their aqueous solutions combining experimental methods (Section 3.2.2) and simulation techniques (Section 3.2). Steady shear rheological measurements were used to assess the effect of the concentration and HEUR structure, complemented by extensive coarse-grained MD simulations. These simulations quantify the self-assembly process of HEUR molecules as a function of their structure and thickener concentration using metrics such as the hydrophobic cluster volume, the total micellar volume, the aggregation number (*N*_agg_), and the number of interconnecting chains (*N*_bridged_) with another hydrophobic cluster. In Section 3.3, we integrate the aqueous HEUR solutions from Section 3.2.1 into waterborne latex-based emulsions. We examine the effects of HEUR’s hydrophilic and hydrophobic segments on emulsion viscosity and underscore the critical role of HEUR’s hydrophilic length in the emulsion’s phase stability. Finally, in Section 3.4, we extend our investigation to waterborne paint formulations, specifically examining how the HEUR structure influences the balance between Newtonian and pseudoplastic rheological behaviors. Given that real-world dispersions typically use a mix of HEUR thickeners, identifying optimal structures for leveling and sagging is crucial. For this reason, we link the findings to this particular paint performance. Overall, our research probes how HEUR’s chemical composition impacts rheological behaviors across diverse waterborne dispersions, ranging from aqueous solutions to waterborne coatings.

## 2 Materials, Methods, and MD Simulations

### 2.1 Materials

Polyethylene glycol of molecular weight 8000 g/mol with purity of >99.5% was provided by Clariant. H_12_MDI (4,4-methylenebis(cyclohexyl isocyanate), mixture of isomers, 90% purity from Acros Organics) and 1-octanol (99% purity) from Alfa Aesar were used as received. Bismuth carboxylate (KKAT XCB221), provided by King Industries, was used as the catalyst. Chloroform (>99.8% purity) stabilized with amylene was purchased from Fisher Chemicals and was dried using 4 Å molecular sieves. Acrylic polymer latex emulsion (the solid content is 50%, the particle size is 200 nm, and η_Latex_ = 60 mPa · s measured at 23 °C with a Brookfield viscometer) and the satin paint base were provided by the Arkema group. All reagents were analytical grade and used without further purification.

### 2.2 Synthesis of HEUR-X and Formulation in Water

The one-step HEUR synthesis was performed in bulk from the reaction of PEG, a diisocyanate, and a monoalcohol. For all cases, 250 g of PEG was initially melted in a conventionally heated reactor where a vacuum pretreatment step was applied limiting the moisture of PEG to 500 ppm. In all experiments, the reaction temperature was 85 °C, the reaction time was 60 min, and the catalyst concentration was set to 0.01% based on the total mass of the reactive mixture. Based on the findings of our previous work,^41^ for the one-step HEUR synthesis, we utilized an HMDI/PEG ratio of 1.5 and octanol/PEG ratio of 1. At this reaction stoichiometry, our one-step synthesis maximizes HEUR molecular weight by effectively tripling the molecular weight of the utilized PEG, ensuring complete end-capping. After completion of the polymerization, the entire polymer content of the reactor was diluted with water in 20% w/w solutions by adding water to the reactor without carrying out prior purification steps in the polymer melt. The water-based HEUR formulations were obtained by stirring the mixture overnight, which resembles an industrial practice for the formulation of a thickener product.

### 2.3 Preparation of the HEUR-Latex Based Emulsion and Full Paint Formulation

Latex-HEUR-X dispersions were prepared by following a precise recipe. Initially, 161 g of latex was subjected to gentle homogenization using a submerged stirrer. Subsequently, 45 g of distilled water was added to the mixture. The pH of the dispersion was carefully adjusted by dropwise addition of 28% ammonia solution until it reached a target range of 8.5 to 8.8. Following pH adjustment, 24 g of a 20% aqueous solution of HEUR was introduced to the dispersion, and the mixture was vigorously stirred at a speed of 1100 rpm for 15 min. Upon completion of the mixing procedure, the resulting mixture was allowed to equilibrate under ambient conditions for 2 days before further testing.

A satin paint with a pigment volume concentration (PVC) of 32.28 was chosen for the thickener performance study. The formulation of the paint is presented in Table 1. The preparation of the paint base was performed by introducing in a suitable container, water, two dispersing agents, a defoaming agent, a neutralizing agent (NH_4_OH (28%)), a biocide, a pigment, and a filler (TiO_2_ and CaCO_3_ size <1 μm). The container was then subjected to strong agitation at 1000 rpm for approximately 15 min to break up filler agglomerates and achieve good dispersion. To monitor the grinding of the paint, a fineness gauge was used to measure the size of individual particles after dispersion. Stirring was continued until the size of the agglomerates was below 20 μm. Once the desired particle size was achieved, the remaining binder, two coalescing agents, water, and a defoaming agent were added to the mixture. The formulation was left under vigorous stirring for 1 h before the addition of the thickener. The full formulation of the paint was stirred until homogenization was achieved.

**Table 1:** Generalized Paint Formulation Used and Its Chemical Nature for the Present Study

**name of ingredients**	% wt	**chemical nature**
water	12.0%	
dispersing agent	0.6%	potassium based polyacrylate salt
defoaming agent 1	0.2%	polyether siloxane
neutralizing agent	0.1%	NH_4_OH (28%)
biocide	0.2%	2-methyl-2*H*-isothiazole-3-one and 1,2-benzisothiazol-3(2*H*)-one
pigment	18.8%	TiO_2_
filler	13.0%	CaCO_3_ < 1 μm
binder	41.6%	styrene acrylic
coalescing agent 1	1.0%	monopropylene Glycol
coalescing agent 2	1.0%	ester alcohol
water	3.3%	
defoaming agent 2	0.2%	polyether siloxane copolymer
paint base	**92%**	
thickener in water 20% w/w	4.0%	HEUR
water	4.0%	
total paint	**100%**	
thickener dry content (% w/w)	0,8%	

### 2.4 Analytical Methods

#### 2.4.1 Gel Permeation Chromatography (GPC)

The weight-average molecular weight (*M*_w_) and the number-average molecular weight (*M*_n_) were determined by GPC from Shimadzu using four Styragel columns from Waters. The polydispersity index, PDI, was calculated as PDI = *M*_w_/*M*_n_. Chloroform was the mobile phase (1 mL/min) at a 30 °C operating temperature. Polyethylene glycol/oxide (PEG/PEO) standards were used for calibration. The samples were collected based on the “in situ” method,^42^ in which the molten samples were directly dissolved in vials with preweighed dry chloroform.

#### 2.4.2 Fourier Transform Infrared Spectroscopy (FTIR)

The qualitative analysis of the obtained polymers was performed using attenuated total reflectance-Fourier transform infrared spectroscopy (ATR-FTIR, PerkinElmer, Spectrum 100, USA). At least four scans for each sample were conducted in the span range of 4000–650 cm^–1^. The samples were again collected based on the “in situ” method.^42^ The liquid samples were placed in the analysis cell, and the spectra were recorded after total spontaneous solvent evaporation.

#### 2.4.3 Rheological Measurements

The rheological properties of HEURs in aqueous solutions were measured with a HAAKE/MARS iQ Air rheometer using a cone and plate geometry of 35 mm diameter, 2° angle cone (C35 2.0°/Ti). The distance of the gap was 0.096 mm. Water-HEUR solutions were prepared as described above (Section 2.2). The water amount was selected based on industrial tests performed during the commercialization stage of a thickener product. Seventeen to 20% dilution in water is normally applied and considered representative of the downstream processing behavior of the final product. The zero-shear viscosity and shear stress profiles were obtained for shear rate testing from 0.01 to 1000 s^–1^. Two types of oscillatory experiments were conducted: (1) frequency sweep at a constant strain and (2) strain sweep at a constant frequency of 1 Hz. The strain sweep experiments were performed initially to determine the critical strain value for each sample, which signifies the point at which the sample structure begins to break down. A strain value below the critical strain was subsequently utilized in the frequency sweep experiments. Three-interval thixotropy tests (3ITTs) were performed to evaluate the thixotropy of the samples. All rheological measurements were performed at 23 °C.

#### 2.4.4 Antisag Index (ASI) Determination

ASI was determined with a BYK-Gardner Anti-Sag Meter (BYK-Gardner USA) following the procedure of ASTM D4400–18.^9^ The applicator contains multiple notches with varying clearances spanning 3–12 or 4–24 mil. Each notch is 1/4″ (6.4 mm) wide and separated by 1/16″ (1.6 mm) spacing. Approximately 10 mL of freshly presheared paint was transferred onto a Leneta Form 9A opacity-display test chart (Leneta, USA), which is made of paper characterized by a black and white, sealed, and smooth surface. Then, the multinotched applicator was drawn down across the chart, which generated a series of evenly spaced stripes. The chart was then promptly hung vertically and allowed to dry at room temperature. After drying, samples were inspected visually and rated for an ASI, which is defined as the clearance of the gap that produces the thickest film stripe not sagging completely to the stripe below.

#### 2.4.5 Determination of the Flow-Leveling Performance of the Paint

Leveling assessments were performed using a Leveling Applicator LTB-2, which adheres to the guidelines outlined in ASTM D4062, the American standard for evaluating leveling properties. This specialized applicator consists of a threaded stainless-steel rod that functions as a grooved doctor blade, enabling the creation of a film with parallel ridges and valleys to simulate brush marks. The LTB-2 features alternating clearances of 300 and 100 μm, allowing for the application of stripes with thicknesses of 150 and 50 μm, respectively. The resulting wet film thickness of the test drawdown was approximately 100 μm. To assess leveling, three-dimensional plastic cards representing various levels of leveling were utilized, ranging from extremely poor (card 1) to excellent (card 9). This standardized approach using the Leveling Applicator LTB-2 and the plastic cards provides an objective and reliable means for evaluating paint leveling, yielding valuable insights into the performance and quality of the tested coatings.

#### 2.4.6 Accelerated Aging Test

An accelerated test used to predict the thermal stability of coatings with time was performed. The test generally involves measuring the viscosity change after the paint has been heat-aged for a week at 50 °C. This test simulates the stability of the paint over a 6 month period. The effect of the loss of viscosity of these samples on the long-term thermal stability was studied.

### 2.5 Coarse-Grained Molecular Dynamics Simulation

**Scheme 1:**
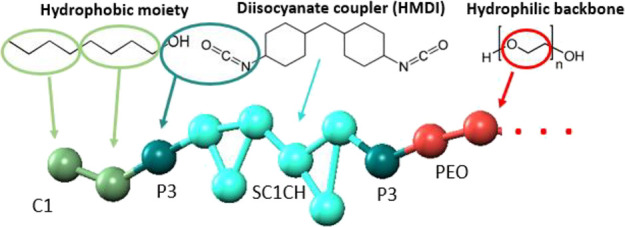
Mapping from Atomistic to Coarse-Grained Representation Using Martini Beads The schematic illustrates only one end (left side) of the polymer chain.

Coarse-grained molecular dynamics simulations were performed utilizing the MARTINI framework and force-field.^43^ A schematic illustration of the mapping from atomistic to coarse-grained representation is shown in Scheme 1. A four-to-one mapping was used; i.e., on average, four heavy atoms and associated hydrogens were represented by a single bead. Four carbons in the indicative octanol molecule were grouped into one C1 bead.^43^ For hexanol as the hydrophobic moiety, small Martini beads SC1 were used for grouping three carbons into one bead. Small beads denoted with “S” have reduced interactions; i.e., the epsilon of the Lennard–Jones potential is scaled to 75% of the original value, and sigma is set to 0.43 rather than 0.47 nm.

The hydroxyl reacting with the isocyanate group were mapped onto one P3 bead.^43^ The cyclohexane rings were modeled by three connected beads, noted here as SC1CH. They are SC1 beads with bonding parameters taken from the Martini site,^43^ suitable for cyclohexane. The hydrophilic segment of HEURs was represented by a chain of PEO beads adopting the parametrization presented elsewhere.^43^ Each PEO monomer was mapped onto one CG PEO bead. The PEO polymer chain ends on both sides with the same sequence of beads as shown in Scheme 1 for the left side. Finally, water was modeled with one P4 bead that groups four water molecules.^43^

All systems were created and simulated using the MAPS platform (Materials and Processes Simulations Platform, v. 4.5, SCIENOMICS SAS, Paris, France). The initial configurations were produced by randomly mixing the components (polymer chains and water) at a density of 0.8 g/cm^3^ using the Amorphous builder of MAPS and subsequently energy minimized using LAMMPS (version 29 Sep 2021). Seventy polymer chains with 362 PEO beads (corresponding to a MW equal to 16,000 g/mol) were placed in water so that the composition of the system was equal to 10, 20, or 35 wt % in polymer. The number of water beads was in the range 60,000 to 75,000, whereas the box length was between 210 and 250 Å. For validation purposes, all simulations presented in this work were repeated five times, starting with different random initializations.

The simulations were performed with LAMMPS at the NPT ensemble for 500 ns at ambient temperature and pressure with a time step of 5 ps. The Gromacs pair style of LAMMPS was used with a cutoff of 12 Å and switching at 9 Å.

#### 2.5.1 Automated Micelle Identification and Quantification

In our study, we employed machine learning (ML) techniques to automate the identification and quantification of micelles. This process eliminates the need for manual micelle counting and enables the determination of average aggregation numbers and average micellar volumes across multiple frames. Specifically, we utilized the unsupervised ML algorithm known as DBSCAN (Density Based Spatial Clustering of Applications with Noise)^44^ that is integrated into the Analysis Tool of the MAPS platform.

DBSCAN relies on two key parameters: the minimum distance for seeking neighboring beads (the ε parameter of DBSCAN, set to 10 Å in our computations and is approximately twice the maximum bond length between beads) and the minimum number of beads required to form a cluster. We set the latter threshold equal to the number of hydrophobic beads per molecule, denoted as minPts. DBSCAN scans each bead within the MD system and retrieves its Cartesian coordinates in 3D space. Each bead is deemed a cluster bead if there exist at least minPt beads within a defined distance, ε. Beads situated within the ε distance of a core bead but do not have at least minPts in their neighborhood are classified as border beads and thus belong to a cluster. Noise beads are neither core nor border beads and do not belong to any cluster. DBSCAN explores the ε neighborhood of a bead to identify cluster beads and augments the cluster by adding core beads and their neighbors (within an ε radius), and this process continues until no more core points can be added. This automated approach facilitates the identification of the hydrophobic core around which a micelle forms. Utilizing MAPS’ visualization tools that enable different coloring of identified clusters, we visually validated the findings of the DBSCAN algorithm. We leveraged the MAPS Analysis Tool’s capabilities to measure the volume of each hydrophobic core. In particular, we employed Monte Carlo based techniques for calculating the volume of irregularly shaped objects^45,46^

Once we identified the hydrophobic core for each micelle, we proceeded to identify the HEUR chains attached to it and subsequently calculated (again through Monte Carlo techniques) the volume of each micelle. As part of our analysis, we also identified the bridge chains that connect different micelles to one another.

## 3 Results and Discussion

### 3.1 Structural Characterization of HEURs

**Scheme 2:**
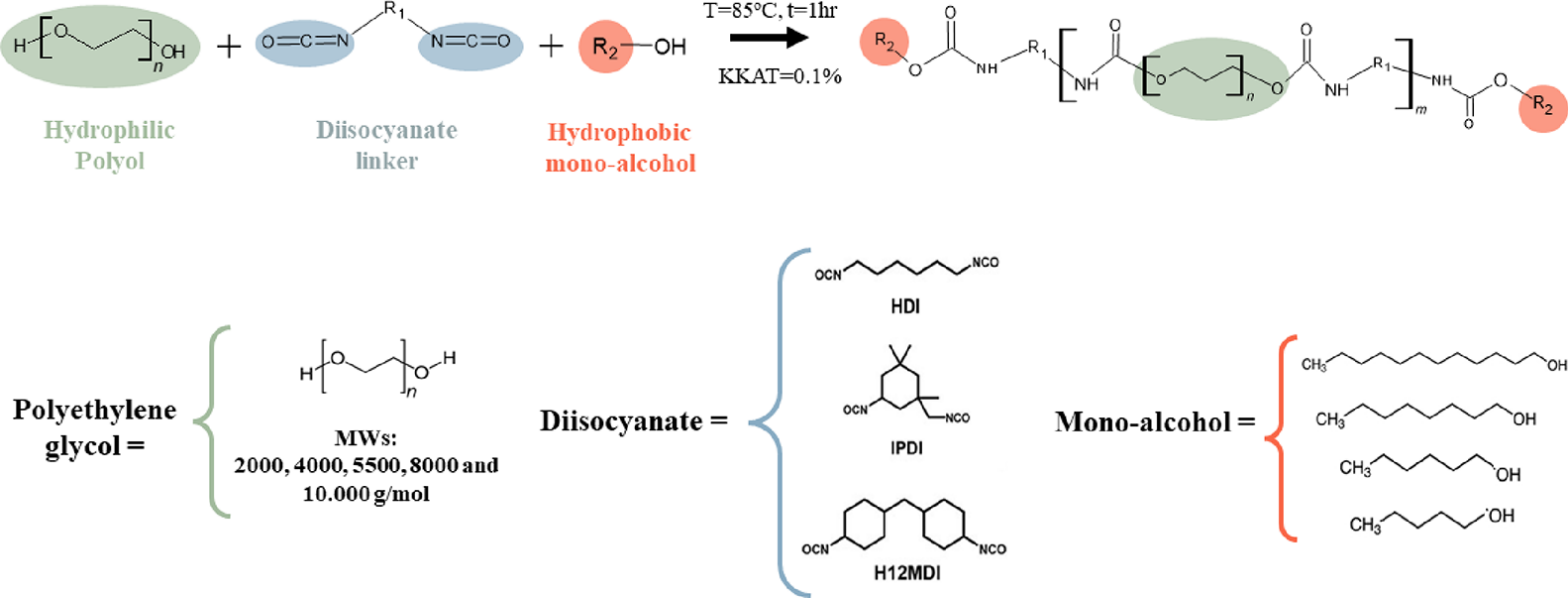
Reaction Scheme and Structure of Reactants Used in This Study

The synthesized HEURs, employing various PEGs, diisocyanates, and monoalcohols, are detailed in Table 2, whereas the one-step synthesis route and corresponding HEUR structures are outlined in Scheme 2. To assess the individual influence of each segment on the rheological behavior of HEUR formulations, we adhered to the principle of ceteris paribus, systematically modifying a single segment in each experiment while other structural factors remained constant. GPC measurements, shown in Table 2, revealed that the number-average molecular weight (*M*_n_) and the polydispersity index (PDI) of HEUR were effectively controlled. Given the consistent reaction conditions and stoichiometry across all experiments, the *M*_n_ remained almost the same (≈23,000 g/mol) when varying the diisocyanate and monoalcohol structures. By adjusting the molecular weight of the PEG, we effectively altered the resultant molecular weight and, consequently, the hydrophilic length of HEUR, tripling the PEG’s molecular weight in each case. In the rest of the paper, the term “molecular weight” refers to *M*_n_, although this notation will not be used for the sake of simplicity.

**Table 2:** Structural Segments of HEUR, Molecular Weights (determined by GPC), and PDI Values

	HEUR	hydrophilic segment	diisocyanate linker	hydrophobic segment (monoalcohol)	*M*_n_ (g/mol)	PDI
hydrophobic segment	HEUR-5: P8-HDI-C8	PEG 8000	HDI	1-octanol	27,100	1.8
	**HEUR-6: P8-IPDI-C8**	PEG 8000	IPDI	1-octanol	23,200	1.7
	**HEUR-7: P8-HMDI-C12**	PEG 8000	HMDI	1-dodecanol	22,500	1.7
	**HEUR-1: P8-HMDI-C8**	PEG 8000	H_12_MDI	1-octanol	23,200	1.7
	**HEUR-2: P8-HMDI-C6**	PEG 8000	H_12_MDI	1-hexanol	22,300	1.7
	**HEUR-10: P8-HMDI-C5**	PEG 8000	H_12_MDI	1-pentanol	22,500	1.7
hydrophilic segment	**HEUR-9: P2-HMDI-C8**	PEG 2000	H_12_MDI	1-octanol	8400	1.8
	**HEUR-7: P4-HMDI-C8**	PEG 4000	H_12_MDI	1-octanol	14,000	1.7
	**HEUR-3: P5.5-HMDI-C8**	PEG 5500	H_12_MDI	1-octanol	17,800	1.7
	**HEUR-4: P10-HMDI-C8**	PEG 10,000	H_12_MDI	1-hexanol	32,700	1.8

**Figure 1:**
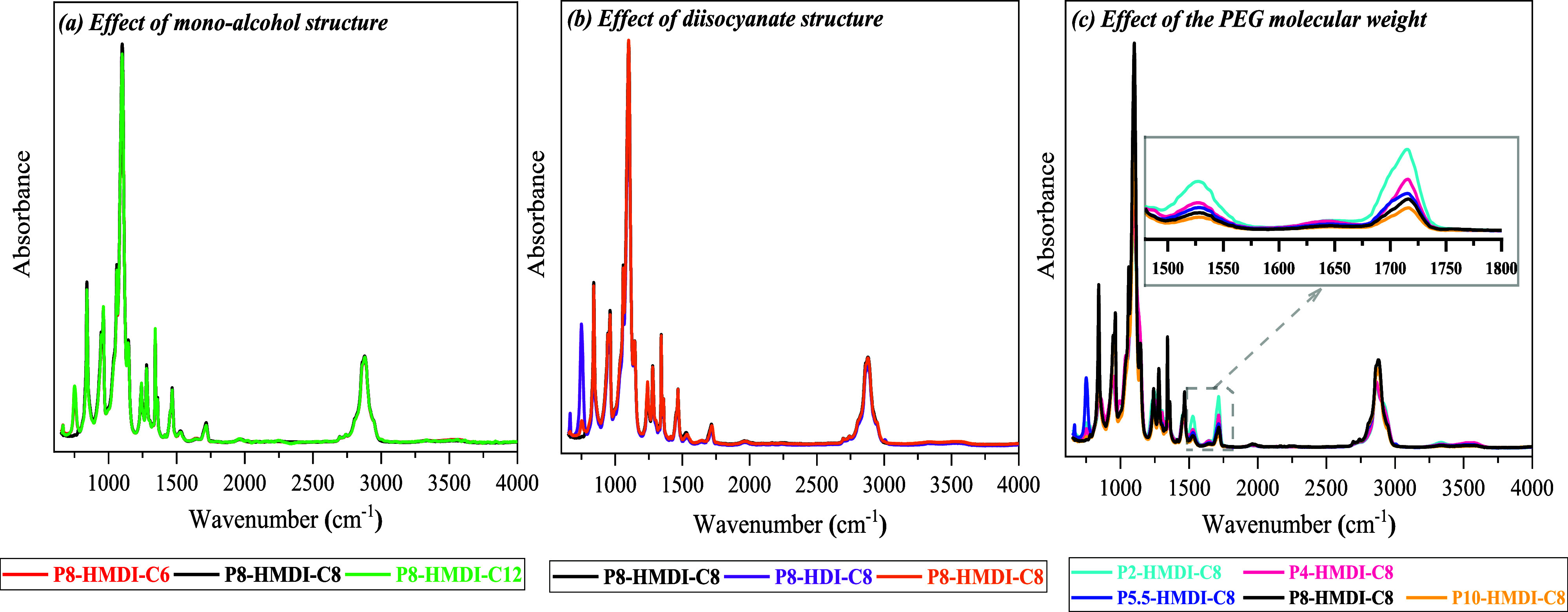
FTIR spectra of HEURs synthesized with different (a) monoalcohols, (b) diisocyanates, and (c) PEG’s.

FTIR tests were performed to determine the chemical composition of the produced HEUR thickeners and to compare the structure–property relationship with those of the structural segments of HEUR. Figure 1 shows the FTIR spectra of 10 HEUR thickeners synthesized with different (a) monoalcohols, (b) diisocayantes, and (c) molecular weights. For all analyzed samples, the characteristic absorption bands of polyethylene glycol appear in the range 840–1466 cm^–1^,^47,48^ and the characteristic −CH stretching band appears in the range 2700–3000 cm^–1^. Considering that the characteristic absorption peak of the isocyanate group (N=C=O) at 2265 cm^–1^^42,48,49^ is absent for all samples, complete end-capping is ensured. Additionally, for all samples, characteristic urethane peaks appear at 1715 and 1530 cm^–1^ that can be assigned to the disordered hydrogen-bonded carbonyl (C=O) groups^42,50−53^ and the bending vibrations of the NH^42,48,49,51,54^ in the urethane polymers. The peak at 1640 cm^–1^ is attributed to the traces of ordered hydrogen-bonded urea carbonyl (C=O).^42,48,49,51,54^ Based on the spectra obtained, no structural differences are observed when the monoalcohol and diisocyanate structure is varied, which is also verified by the identical molecular weights measured with GPC. However, when varying the molecular weight of PEG and, as a consequence, the molecular weight of HEUR, differences can be observed in the intensity of the peaks appearing in the range of 1500–1700 cm^–1^; these can be attributed to the urethane bond as previously mentioned. The intensity of these peaks is notably more pronounced for the HEURs synthesized using lower-molecular-weight PEGs because of the relative prominence of the urethane bond’s signal when a lower-molecular-weight PEG is used.

### 3.2 Impact of the Chemical Structure of HEURs on Micelle Formation and on the Rheological Behavior of Their Aqueous Solutions

**Figure 2:**
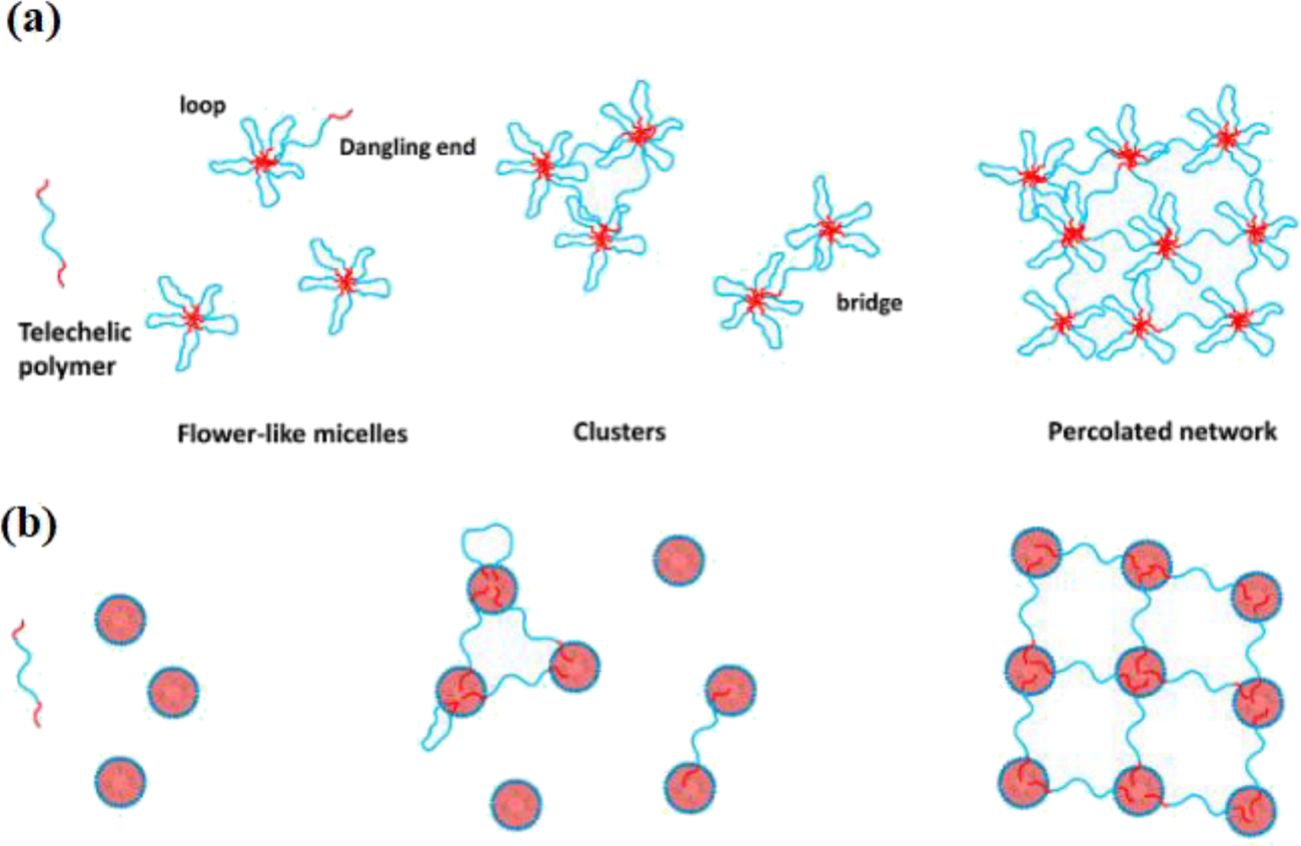
Schematic representation of (a) the association of HEURs in aqueous solutions and (b) with latex-based emulsions as a function of the polymer concentration, redrafted from ref (60) that has been licensed under an open access Creative Commons CC BY 4.0 license.

Aqueous HEUR solutions, dependent on polymer concentration, can form distinct micelles characterized by hydrophobic cores and water-soluble PEO corona^1,11,26,27,55,56^ (e.g., Figure 2). Flower-like micelles are formed above a relatively low concentration of HEURs in water, generally between 0.1 and 4% w/w (depending on the polymerization methodology).^10,11,25,57,58^ At these concentrations, the viscosity remains relatively low. However, as HEUR concentration increases, a marked increase in viscosity is observed as a result of the the interconnection of bridged clusters, indicating the onset of the percolation transition and the formation of a transient network.^1,25,58,59^ The higher the number of bridges between micelles is, the higher the network density and, consequently, the solution viscosity are. The network density and therefore the rheological properties of HEURs mainly depend on their concentrations and chemical composition. Albeit HEUR thickeners exhibit application-oriented performance at concentrations (20% w/w) far exceeding their documented CMC regime, the percolation transition effects, which are responsible for their rheological performance, are understudied. Additionally, there have been many attempts in the literature to understand and predict the impact of hydrophilic and hydrophobic segments of HEURs on the rheology of their aqueous solutions, yet some findings remain controversial, likely owing to the different synthetic methods applied that influence the end-product behavior.

Our study employs the same synthesis method for all HEUR structures to evaluate the impact of both the HEUR concentration and its chemical composition on aqueous rheology. In Section 3.2.1, we examine the role of HEUR’s hydrophilic and hydrophobic segments in its aqueous thickening behavior and establish the link between the percolation transition and HEUR’s molecular structure. Additionally, we study the rheological behavior of 20% w/w HEUR aqueous solutions, a critical concentration for paint manufacturers, to identify HEUR structures exhibiting Newtonian, balanced, or pseudoplastic behavior in water.

Our experimental findings are complemented by coarse-grained molecule dynamics (CGMD) simulations (see Section 3.2.2). This approach enables us to investigate the morphology and structural conformation of micellar clusters as a function of HEUR concentration and chemical structure. CGMD simulations are particularly well-suited for the study of complex molecular systems including biomolecules and polymers. In comparison to atomistic simulations, they are computationally less demanding, allowing us to explore larger and longer time-scale systems. The initial configurations are generated by randomly mixing polymer chains and water, mimicking the initial state of the mixture in our experiments. Given that micelle formation is a process that unfolds over large time scales, conducting simulations at the atomistic level becomes impractical. In contrast, CGMD provides a more efficient sampling of the conformational space facilitating the exploration of the energy landscape of a system and the discovery of phase transitions while significantly reducing computational costs. This hybrid methodology, combining both macroscopic and microscopic analyses, offers a new perspective that has not been provided in previous studies.

#### 3.2.1 Structure–Concentration Impact on the Rheological Properties of Aqueous HEUR Formulations: Experimental Study

**Figure 3:**
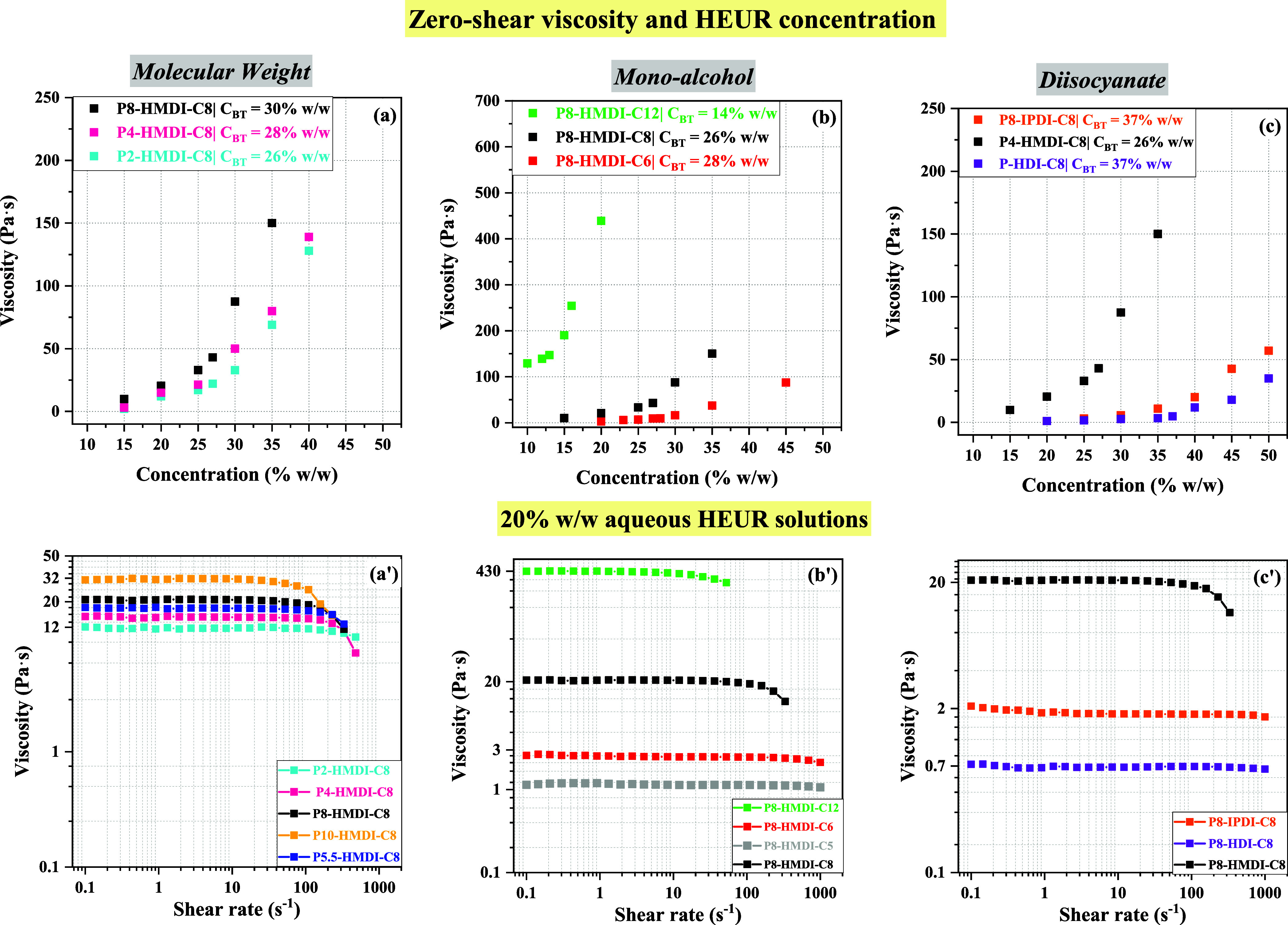
Upper row: correlation between zero-shear viscosity and HEUR concentration; bottom row: steady shear viscosity curves for the 20% w/w HEUR aqueous formulations. (a, a’) Impact of PEG molecular weight; (b, b’) influence of monoalcohol length; and (c, c’) effect of diisocyanate structure. Labels contain the measured *C*_BT_ values for each HEUR tested.

Figure 3 illustrates the relationship between viscosity, HEUR concentration, and composition. Specifically, Figure 3a–c in the top row displays how zero-shear viscosity correlates with HEUR concentration for different chemical architectures. The results show that the influence of HEUR concentration on viscosity is subtle at low concentrations, but beyond a certain threshold, viscosity rises sharply for all HEUR structures. This threshold, termed the critical bridging threshold (*C*_BT_) in our study, signifies the point at which bridging markedly affects viscosity. For each HEUR examined, the *C*_BT_ was calculated by fitting the viscosity–concentration data with two linear curves, one for low and another for high concentrations. The intersection of these curves denotes the *C*_BT_. All determined *C*_BT_ values are indicated in the labels within Figure 3a–c.

The bottom row, Figure 3a’–c’, presents steady shear viscosity curves for 20% w/w HEUR aqueous formulations. The behavior of specific HEURs, namely, P10-HMDI-C8, P5.5-HMDI-C8, and P8-HMDI-C5, is also shown for comparison, even though their respective *C*_BT_ values have not been determined.

The data presented in Figure 3 show that viscosity varies depending on the diisocyanate structure and monoalcohol length. For instance, the bulkier and more hydrophobic diisocyanate H_12_MDI (with two cyclohexane rings) yields viscosities higher than those of IPDI (with a single cyclohexane ring) and HDI (linear structure) across all tested concentrations. HEURs modified with IPDI and HDI exhibit higher *C*_BT_ values of around 37% compared to HMDI’s 26%, indicating that the HMDI-modified HEUR forms a denser network due to its higher hydrophobicity.

Monoalcohol length also influences viscosity and *C*_BT_ values. As we transition from C6 to C8 and then to C12, solution viscosity increases with C12 presenting notably higher viscosities and lower *C*_BT_ values. The latter indicates that the incorporation of C12 as terminal monoalcohol promotes very strong hydrophobic associations, and the higher viscosity values can been ascribed to the enlarged hydrophobic micellar clusters and the slower motions of individual polymer chains as the residence time of the hydrophobic tails in “polymer micelles” increases.^26,27,57^ Generally, a shift toward longer relaxation times is expected when the polymer chains in a solution become more entangled (due to high polymer concentration, increased hydrophobicity of the polymer’s tail, or higher polymer molecular weight). Figure 3a’,b’ illustrates that HEUR solutions with less hydrophobic terminal hydrophobes, such as P8-IPDI-C8, P8-HDI-C8, P8-HMDI-C5, and P8-HMDI-C6, exhibit a Newtonian behavior and low viscosities across all tested shear rates. In contrast, HEURs with more hydrophobic terminal groups display increased viscosities and a pseudoplastic behavior.

We further explored the impact of HEUR’s hydrophilic length by adjusting its molecular weight from 8000 g/mol (P2-HMDI-C8) to 33,000 g/mol (P10-HMDI-C8), a range selected because of its industrial relevance. As illustrated in Figure 3a, although there is a discernible increase in viscosity with increasing HEUR molecular weight across all tested concentrations, these increments are subtle, especially when contrasted with the pronounced influence of the hydrophobic segment. Additionally, as the molecular weight increased from 8000 to 23,000 g/mol, there was a modest reduction in *C*_BT_ values from 30 to 26% suggesting that a denser transient network formed with higher-molecular-weight HEURs. The steady shear analysis for the 20% w/w HEUR aqueous solutions reveals that an escalation in molecular weight within this range resulted in slightly enhanced viscosities and an earlier onset of the shear thinning effect. Upon surpassing a molecular weight of 23,000 g/mol and reaching 33,000 g/mol, significantly higher viscosities were observed compared to lower molecular weights. However, the steady shear viscosity profiles for the tested HEUR molecular weights are more balanced rather than showing the Newtonian or pseudoplastic trends determined by the hydrophobic segment of HEUR.

It is expected that as the hydrophilic length of HEUR increases, the size of the loops on the floret-shaped aggregates would increase and less looping chains would be involved in a micelle, leading to a lower aggregation number.^11,57,61^ Larger micelles lead to a viscosity increase due to the increase in the hydrodynamic polymer volume. With low-molecular-weight HEURs, intramolecular associations of hydrophobic groups are favored compared to intermolecular associations because of their proximity. In this context, the observed similarities in *C*_BT_ values and viscosities of the same order of magnitude could be attributed to aggregates of increased but comparable hydrodynamic volume, which in the case of low HEUR molecular weights would consist of a higher number of loop chains in a micelle. These experimental findings are complemented by CGMD simulations that are presented in the next section.

#### 3.2.2 CGMD Simulations for the Characterization of the HEUR Micellar Morphology in Water

In this study, we explore the spontaneous formation of micelles starting from random mixtures of long polymeric chains and water beads. Although similar systems have been studied in previous works,^62,63^ our investigation extends to higher molecular weights with polymeric chains reaching up to 32,000 g/mol. To ensure the representativeness of the micellar distribution upon system equilibration, we selected a substantial number of polymeric chains ranging from 70 to 210 molecules. Notably, in all our simulations, micelles form in a spontaneous manner (in contrast to the study in ref (60) where preassembled flower-like micelles are simulated), and one can observe a diverse range of structural conformations. Another innovative aspect of our study lies in the automated process of micellar identification and characterization, achieved through the utilization of the unsupervised ML algorithm DBSCAN (Section 2.5.1).

##### 3.2.2.1 Micelle Formation Processes and the Impact of HEUR Concentration and Hydrophilic Length on Micelle Formation

**Figure 4:**
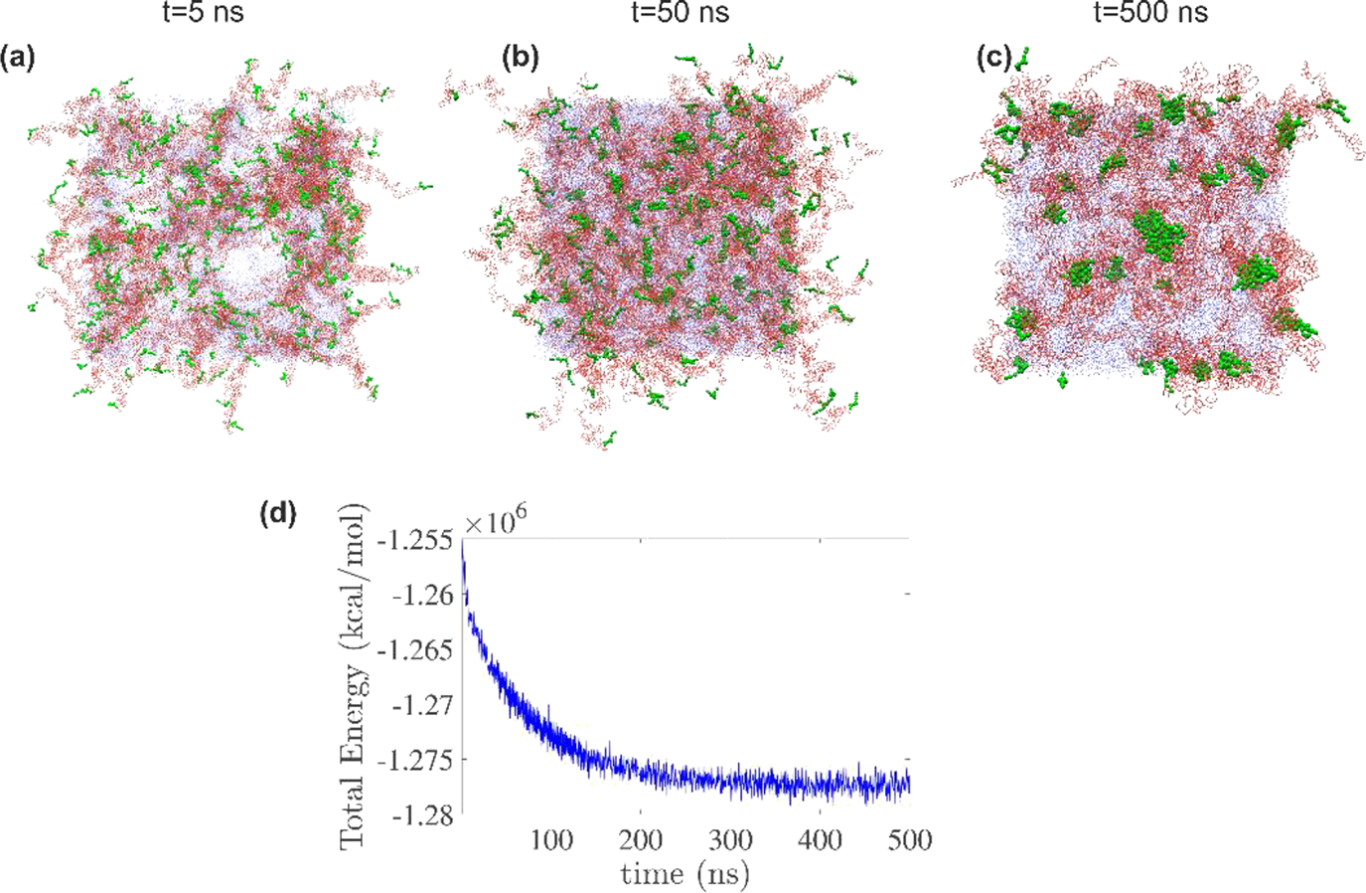
CGMD simulation snapshots of HEUR1/P8-HMDI-C8 in a 20% w/w water solution at *t* = (a) 5, (b) 50, and (c) 500 ns. Color code: blue represents P4 beads (equivalent to four water molecules); red indicates the hydrophilic beads of PEG chains; green spheres represent the hydrophobic end-groups of HEUR. (d) Evolution of the system’s total energy. The energy reaches a plateau (equilibrium) after approximately *t* = 300 ns.

First, we studied the self-assembly of HEUR1/P8-HMDI-C8 in a 20% w/w water solution by analyzing molecular configurations and monitoring the time evolution of the system’s energy to determine its equilibrium state. Figure 4 a,b,c depicts the molecular configurations at *t* = 5, 50, and 500 ns (final structure), respectively. Figure 4d shows the system’s total energy evolution with the system converging to equilibrium after approximately *t* = 300 ns. One can observe the gradual formation of micellar structures, where the hydrophobic end-groups of HEUR (depicted with green spheres in Figures 4a–c) bent inward within the micelle, whereas the hydrophilic PEG chains (depicted in red) remain exposed to the surrounding water (depicted in blue) (Figure 4c). We compute the hydrophobic cluster size distribution by defining a “cluster” as an assembly of terminal hydrophobes excluding the attached PEO chains. Clusters of hydrophobes are identified using the DBSCAN algorithm. Furthermore, we identify the chains attached to these clusters, forming the micelle, and finally, we compute the number of chains connecting two distinct micelles (bridge chains).

**Figure 5:**
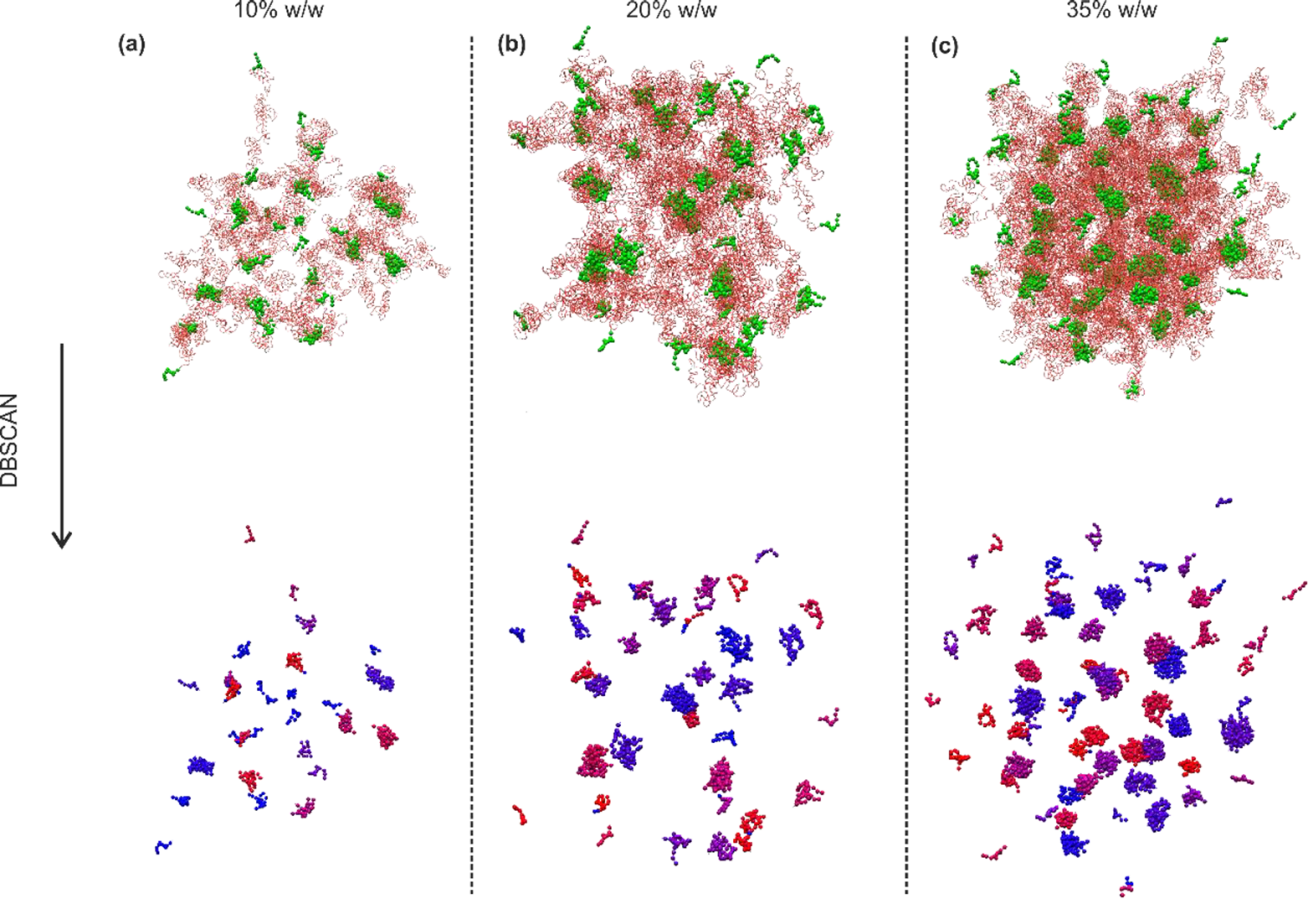
Configuration of the water-HEUR system at equilibrium for (a) 10% w/w, (b) 20% w/w, and (c) 35% w/w concentrations. In the upper panel, PEG chains (in red) and hydrophobic beads (in green) are illustrated; water beads are removed for clarity. The lower panel shows the hydrophobic clusters identified by performing the DBSCAN algorithm (different color for each cluster). The different coloring of clusters is also used for validation purposes of the DBSCAN algorithm.

Figure 5 presents HEUR1 configurations in water at concentrations of 10, 20, and 35% w/w, encompassing concentrations both below and above the experimentally determined critical overlap concentration (Cp) for HEUR1 (26% w/w). The upper layer of Figure 5 illustrates the conformation of hydrophobic clusters and PEG chains (water is omitted for clarity), whereas the lower layer focuses exclusively on the hydrophobic clusters identified by performing the DBSCAN algorithm.

**Figure 6:**
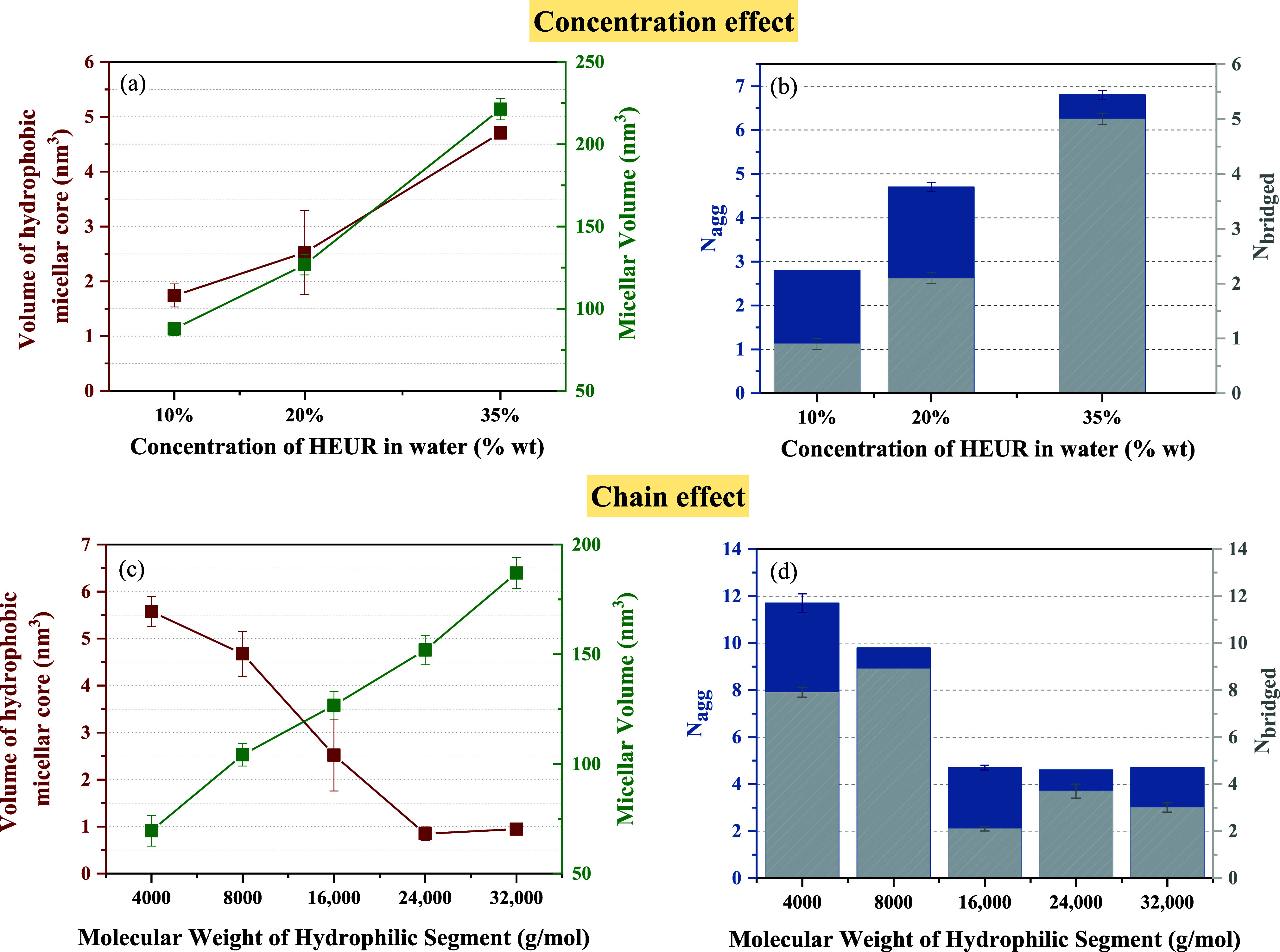
Influence of HEUR concentration and hydrophilic chain length on micellar volume, hydrophobic core volume, and *N*_agg_ and N_bridged_.

Furthermore, Figure 6a,b provides insights into the self-assembly behavior of HEUR at varying concentrations, highlighting key parameters such as (a) the micellar volume (the average total volume occupied by micelles), (b) the hydrophobic core volume, (c) *N*_agg_ denoting the number of chains forming a micelle, and (d) *N*_bridged_ denoting the number of hydrophobic chains within a single cluster that are interconnected with another micelle.

The molecular configurations of HEUR in Figure 5 underscore the concentration-dependent variations in the network density. At low HEUR concentrations, the network appears notably sparse, characterized by fewer and smaller hydrophobic clusters. By increasing the HEUR concentration, the network density enhances, evident in the formation of larger hydrophobic aggregates. This phenomenon is quantified in Figure 6a,b. In particular, Figure 6a illustrates an increasing trend in both micellar volume and hydrophobic core volume as the HEUR concentration increases. Figure 6b confirms this trend by displaying a simultaneous increase in *N*_agg_ and *N*_bridged_ values, indicating both larger hydrophobic clusters and a more interconnected micellar network.

These findings are consistent with the experimental results showing the concentration-dependent viscosity trends for HEUR-1, where higher HEUR concentrations correspond to increased viscosities. In addition, a notable correlation is observed between the experimental critical bridging threshold (*C*_BT_) of HEUR1 and the percentage of bridged/interconnected chains in the micellar network (*N*_bridged_/*N*_agg_), calculated through GCMD simulations. Interestingly, concentrations below the *C*_BT_ (≈26%) exhibit interconnected chain proportions below 45%; however, at 35% w/w HEUR concentration, this percentage sharply rises to 74%, indicating a transition to a densely interconnected micellar network. This transition aligns closely with our experimental data, which show a marked viscosity increase in viscosity when the HEUR concentration surpasses the critical percolation threshold.

Having established that our CG-MD model aligns well with the experimental concentration-dependent viscosity trends observed for HEUR-1, we expanded our study to investigate the impact of HEUR’s hydrophilic chain length on micelle sizes and morphology. Specifically, we performed simulations for HEUR molecular weights ranging from 4000 to 32,000 g/mol, in alignment with available experimental data for molecular weights ranging from 8000 up to 33,000 g/mol.

Figure 6c,d highlights contrasting trends between the impact of HEUR’s hydrophilic chain length and HEUR concentration (contrasting with Figure 6a,b). As HEUR molecular weight increases, the hydrophobic core volume decreases, and fewer HEUR chains participate in micelle formation, as evidenced by the decrease in *N*_agg_ values. In particular, for PEG molecular weights of 4000 and 8000 g/mol, *N*_agg_ ranges from approximately 10 to 12, dropping to around 4.5 for molecular weights between 16,000 and 32,000 g/mol. This decrease in *N*_agg_ and hydrophobic core volume is attributed to increased repulsive interactions between elongated hydrophilic HEUR chains and the larger steric hindrance of hydrophobic groups. Interestingly, despite the lower *N*_agg_ values associated with longer hydrophilic segments, the overall micellar volume continues to expand, aligning with the viscosity trends observed experimentally. Regarding the proportion of interconnected hydrophobic clusters, the data do not exhibit a monotonic trend across varying HEUR molecular weights, making it challenging to definitively assess the influence of molecular weight on inter- and intramolecular interactions.

### 3.3 Effects of Latex and HEUR Chemical Structure on Rheology and Phase Stability of Waterborne Latex–HEUR Mixtures

In waterborne paints, the binder, often termed latex, typically of acrylic or vinyl-acrylic origin, constitutes 15–40% w/w of the formulation and plays a critical role in the overall paint properties. Given its critical role, industry standards dictate that the effectiveness of newly developed associative thickeners, such as HEURs, should be evaluated based on their incorporation into latex-based emulsions.

Latex is a colloidal system in which small hydrophobic polymer particles are dispersed in water. In such system, the hydrophobic segments of HEUR tend to associate with the hydrophobic surface of the latex particles, whereas the hydrophilic segments remain in the aqueous phase.^6,8,12^ When the latex surface is not saturated by surfactants with higher affinity and given sufficient thickener concentration, HEUR molecules can bridge latex particles by adsorbing their hydrophobic segments. The strength of these interactions depends on the chemistry of both the HEUR and latex surface.^6,13,30−37^ Previous studies have identified various association mechanisms of HEUR with latex particles.^6,8,12,13,38,39^ These include a single hydrophobe adsorbed on a latex particle with another bridged on a HEUR micellar cluster, adsorption on different latex particles forming bridges, or adsorption to the same latex particle forming loops.^6,8,12,13,38,39^ The favored interactions depend on several factors including latex surface polarity, thickener hydrophobicity, temperature, and the concentration of latex and thickener.

To align with industry standards, our study incorporated 20% aqueous solutions of HEUR in latex emulsions, with a formulation consisting of 70% w/w latex, 2.09% HEUR, and 27.91% water. Notably, mixtures containing lower-molecular-weight HEURs (P2-HMDI-C8-Mn = 8000 g/mol and P4-HMDI-C8Mn = 14,000 g/mol) led to immediate phase separation, known as syneresis, transforming the liquid emulsion into a nonliquid, foamy texture (Figure S1 of the Supporting Information), underlining the importance of the hydrophilic length in stabilizing the emulsion. The rest of the samples did not exhibit phase separation (observed with the naked eye) for more than 1 week.

**Figure 7:**
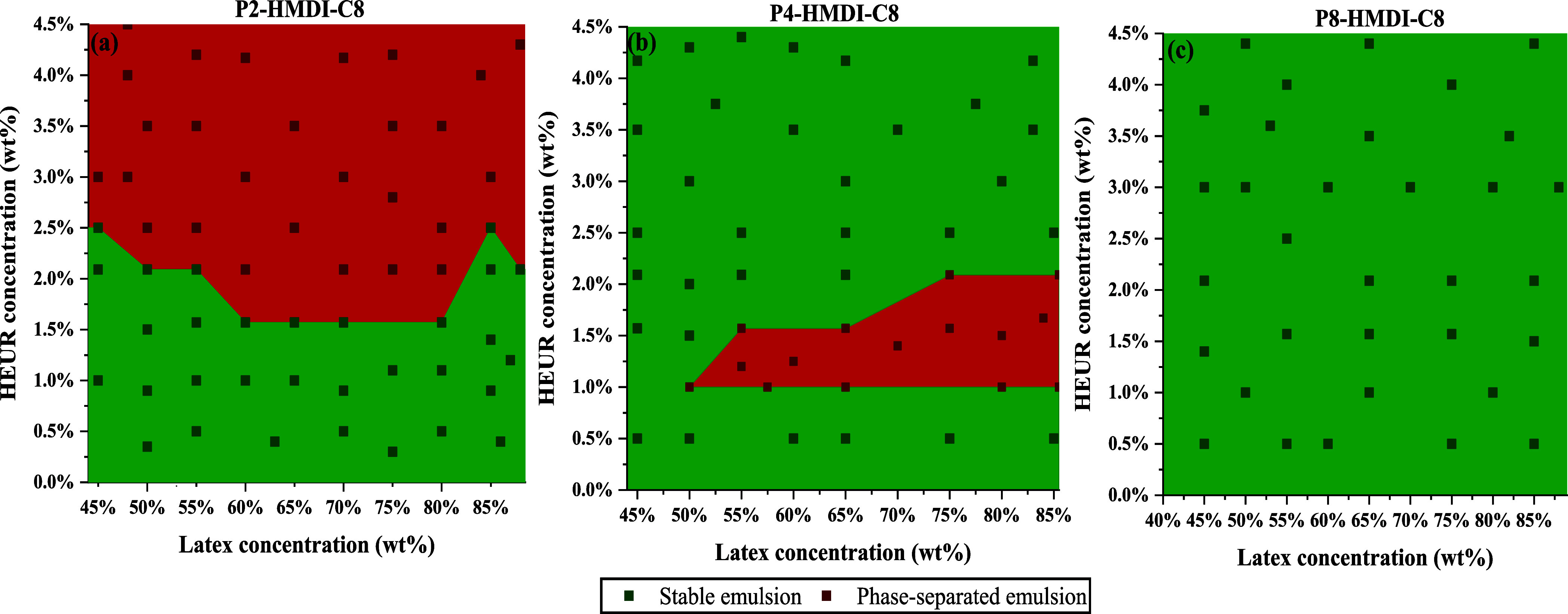
DPDs of waterborne HEUR-latex mixtures of (a) latex 1: formulated with HEUR 8000 g/mol from PEG2000, (b) latex 8: formulated with HEUR 14,000 g/mol from PEG4000, and (c) latex 9: formulated with HEUR 23,000 g/mol from PEG8000. Scatter points indicate the exact formulations tested in our experiments.

**Figure 8:**
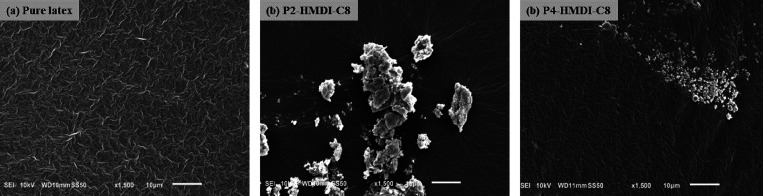
SEM micrographs of the coatings formed from (a) pure latex, (b) latex-based emulsion of P2-HMDI-C8 from the flocculated area, and (c) latex-based emulsion of P4-HMDI-C8 from the flocculated area.

To expand on this analysis, our investigation utilized dispersion phase diagrams (DPDs) initially introduced by Konstansek,^64,65^ who studied the concentration-dependent nature of syneresis in latex-based emulsions featuring a HEUR thickener, surfactant, and latex particles. It is worth noting that the presence of surfactants severely affects the phase separation region, as they can associate with HEURs and the latex surface and finally displace the HEUR hydrophobes from adsorbing to the latex particle surfaces. Figure 7 reveals distinct stability regions for three HEUR-latex formulations: P2-HMDI-C8, P4-HMDI-C8, and P8-HMDI-C8. The formulation containing P2-HMDI-C8, which has the shortest hydrophilic length, manifested a broad flocculation area (depicted in red) across all tested latex concentrations when the HEUR concentration exceeded 2%. On the other hand, P4-HMDI-C8, featuring a longer hydrophilic segment, displayed a more restricted flocculation region confined to specific HEUR concentrations between 1 and 2% and latex concentrations between 50 and 85%. Notably, P8-HMDI-C8, with the longest hydrophilic segment, showed no observable macroscopic flocculation. Additionally, as evident from the SEM micrographs in Figure 8, the pure latex has a distinct surface, whereas the tested flocculated samples of P2-HMDI-C8 and P4-HMDI-C8 appear to exhibit regions of agglomerates. These findings emphasize the critical role of the hydrophilic segment length in determining emulsion stability as it aids in effective interparticle bridging while simultaneously inhibiting the formation of flocculates.

**Figure 9:**
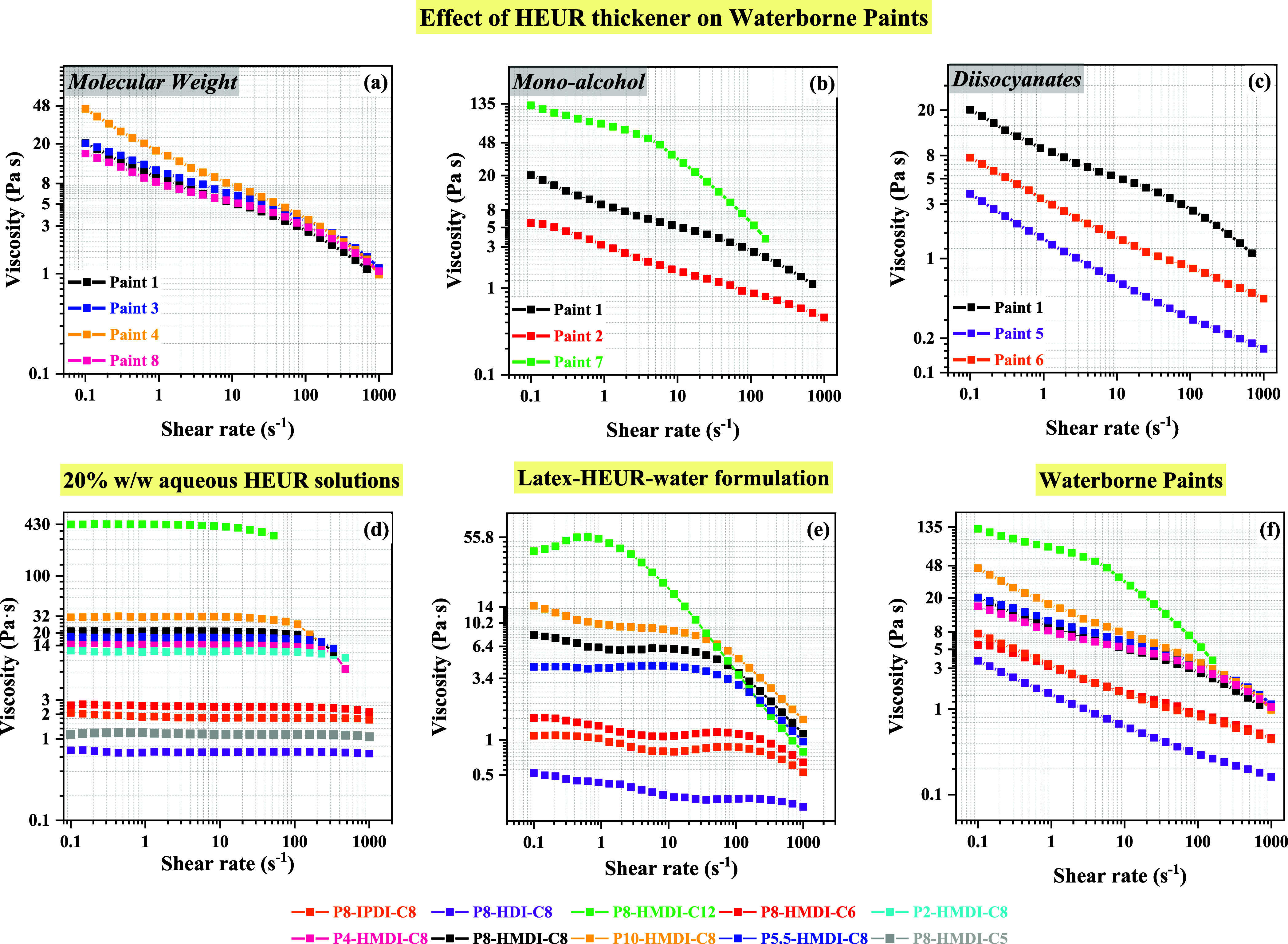
Comparative analysis of steady shear viscosity curves for (upper row) HEUR-thickened paints: (a) influence of PEG molecular weight, (b) impact of monoalcohol length, and (c) effect of diisocyanate structure and for (bottom row) (d) 20% w/w aqueous HEUR solutions, (e) latex-HEUR-water formulations, and (f) waterborne paints numbered according to the numbering of HEURs in Table 2.

For stable emulsions without syneresis, Figure 9d demonstrates the impact of HEUR’s hydrophilic and hydrophobic segments on the rheology of latex-based emulsions. Consistent trends were observed in both the steady shear analysis of HEUR aqueous solutions and the latex emulsions. Enhanced hydrophobicity, attributed to bulkier diisocyanate units or elongated monoalcohol moieties, promotes the formation of a denser transient network, leading to increased emulsion viscosity. Furthermore, when comparing HEURs with identical hydrophobic structures but variable hydrophilic lengths (from 18,000 to 33,000 g/mol), a direct correlation between higher molecular weight and increased viscosity was evident.

### 3.4 Rheological Characterization and Performance Evaluation of Waterborne Paints Thickened with HEURs: Insights from Steady Shear and Oscillatory Rheology, Leveling, Sagging, and Heat Stability Measurements

The aqueous solutions of HEURs were incorporated into waterborne paint formulations to assess the impact of different HEUR chemical structures on the rheological behavior and overall performance of the thickened paints. The rheological characteristics of these paints were analyzed through steady shear viscosity analysis, oscillatory measurements, and 3ITT and thermal stability measurements. Paint performance was further evaluated based on leveling and sagging tests. Detailed results are presented in the following sections.

#### 3.4.1 Steady Shear Analysis

Figure 9a–c depicts a shear-thinning behavior for all paint samples, in line with their characteristic nature as highly solid dispersions. Notably, despite the low concentration of HEURs in the paint formulation (1–3% w/w), modifications in HEUR’s structural segments impact paint viscosity. Remarkably, despite the different association mechanisms of HEUR in paint formulation (multicomponent system) compared to its self-assembly in aqueous solutions (binary system), the results indicate that the rheological behavior of different HEUR structures in aqueous solutions shows the same trend when tested in latex-based emulsions and in the final paint formulation (Figure 9). The Newtonian, balanced, and pseudoplastic behavior was effectively retained when incorporating binder and pigment particles. This consistency indicates that the rheological responses observed in water solutions provide a reliable forecast of their behavior in latex-based emulsions and waterborne paints. Higher viscosity values and a more pronounced thinning effect were obtained when the effective terminal-hydrophobe size of HEUR was increased based on modifications of the diisocaynate and monoalcohol structure accordingly. This effect is also demonstrated in the pseudoplasticity index (PI) values depicted in Table S2 of Supporting Information, where a more hydrophobic terminal tail led to higher PI values. By examining the influence of HEUR’s hydrophilic length, achieved through altering its molecular weight and PEG length, we observe that paints modified with HEURs having molecular weights of 14,000, 18,000, and 23,000 g/mol exhibit similar PI values and flow characteristics throughout the entire spectrum of shear rates. However, extending the hydrophilic length of HEUR further, as demonstrated in the paint modified with a HEUR possessing a molecular weight of 33,000 g/mol, results in higher PI and viscosity values within the low to midshear region, up to 4 s^–1^.

#### 3.4.2 Oscillatory Measurements

**Figure 10:**
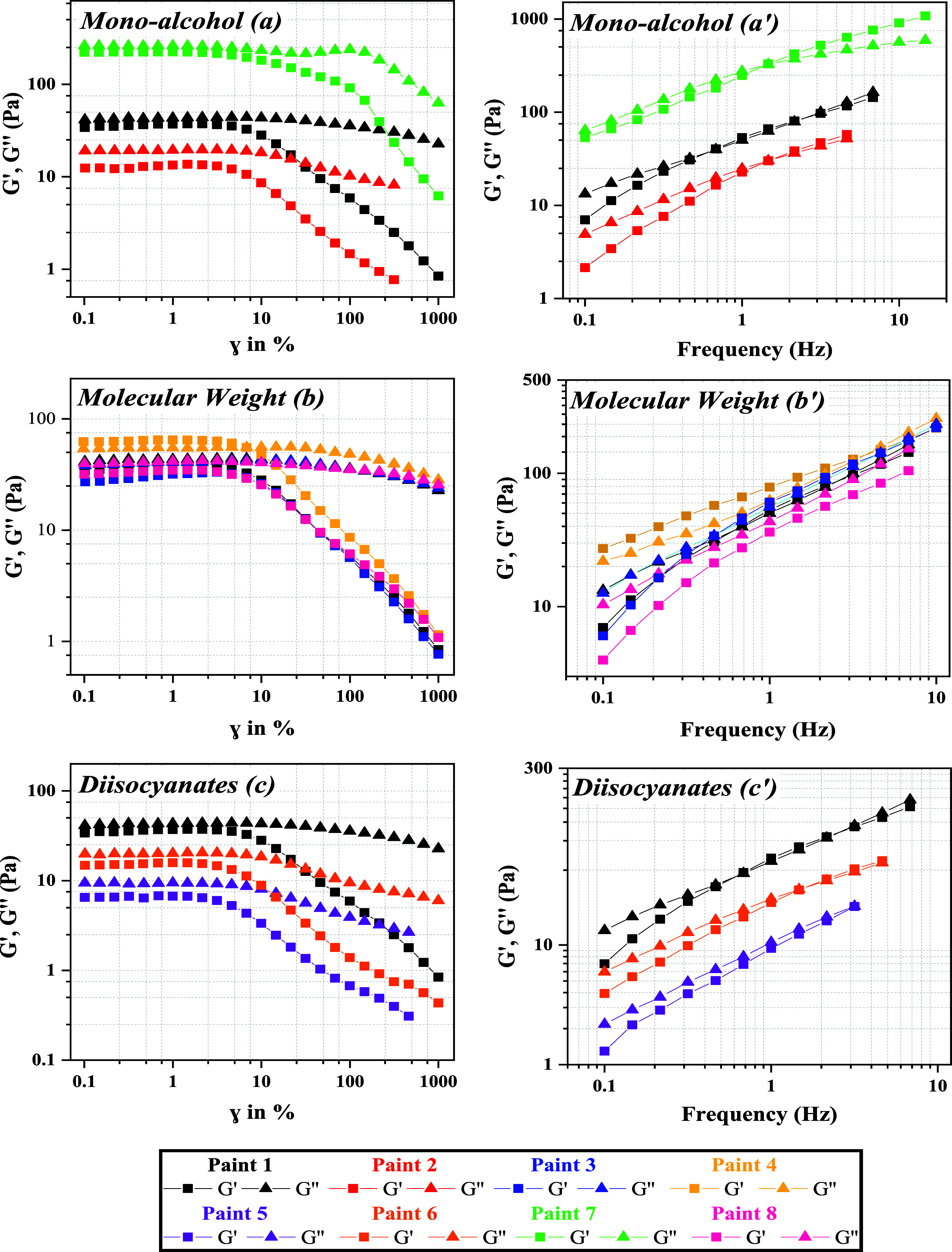
Left panel (a–c): oscillatory strain–sweep curves plotting the elastic (*G*′) and viscous (*G*″) moduli against strain. Right panel (a’–c’): frequency sweep results showing *G*′ and *G*′′ values across a frequency range of 0.1 to 10 Hz conducted at 1% strain.

Paints exhibit complex rheological behaviors that go beyond steady shear analysis, requiring evaluation of their viscoelastic properties to gain insight into associative network strength and pigment dispersion quality. The two common oscillatory tests employed are the amplitude sweep (AS) and frequency sweep (FS). The AS test establishes a linear viscoelastic range (LVER), where the elastic (*G*′) and viscous (*G*″) moduli remain constant, irrespective of strain, at a set temperature and frequency. Subsequently, within this LVER, an FS test examines the paint’s time-dependent properties under minimal stress conditions. Figure 10a–c showcases oscillatory strain–sweep curves with *G*′ and *G*″ plotted against strain. Within the LVER, both moduli exhibit stable plateau values until they reach a critical strain or yield point, after which they decline. Yield values for all paints are determined based on a 5% deviation of *G*′ values^66^ from the plateau values, as summarized in Table S2 of the Supporting Information. Complementary FS results, carried out in the LVER, are shown in Figure 10a’–c’, plotting *G*′ and *G*″ across a frequency range of 0.1 to 10 Hz at a 1% strain.

Upon evaluating the impact of monoalcohol and diisocyanate structure on the frequency and strain-dependent behavior of *G*′ and *G*″, the patterns closely mirror those observed in steady shear analysis. A shift of the AS and FS curves to higher *G*′ and *G*′′ values denotes a more robust associative network, indicative of a superior structural network within the paint’s matrix. This shift is mainly related to interparticle associations, which are strengthened by the incorporation of a bulkier diisocyanate or a longer end-capper into the hydrophobic tail of HEUR. Additionally, the bulkier diisocyanate and the longer monoalcohol generally lead to a lower crossover frequency (*G*′ = *G*′′), illustrating the lower responsiveness of the paint to high-frequency oscillations.

In AS tests, paints with HEUR thickeners of molecular weights between 14,000 and 23,000 g/mol exhibited a liquid-like behavior, as indicated by *G*′′ exceeding *G*′ and similar structural strength (similar *G*′ values). In contrast, a molecular weight of 33,000 g/mol led to a solid-like behavior. FS tests further elucidated these findings. Specifically, paint 8, formulated with the shortest hydrophilic length of HEUR (14,000 g/mol), showed a liquid-like character and lacked a crossover point, indicative of a weaker associative network. Conversely, paints 1 and 3, featuring HEURs with longer hydrophilic lengths, displayed crossover points at similar frequencies, signifying stronger interparticle associations. Most notably, paint 4, containing the highest-molecular-weight HEUR (33 000 g/mol), exhibited a solid-like character.

#### 3.4.3 Connection of 3ITT with Leveling and Sagging

**Figure 11:**
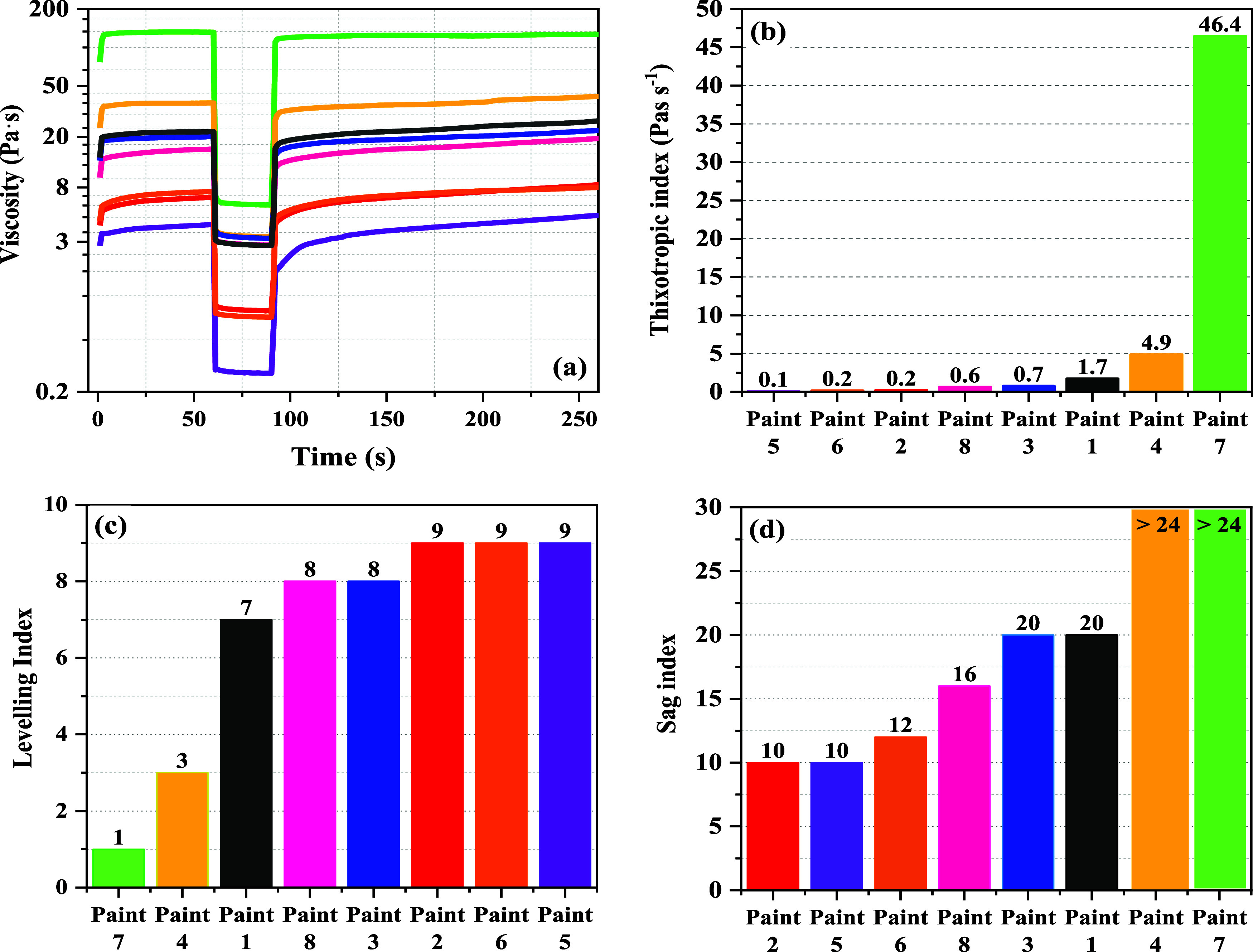
Results of paints 1–8 modified with HEURs 1–8 with varying hydrophilic and hydrophobic structure. (a) 3ITT (structural recovery greater than 100% occurs when the structure is broken down during high shear, which allows for a new arrangement of molecules resulting in a higher structural strength than before the shear load was applied); (b) TI values; and (c, d) results of Leneta chart for leveling and sagging, respectively. Flow leveling index: 0 = very poor and 10 = best, sag index: 4 = poor and 24 = best. The flow leveling and sagging index scale used herein is similar to that employed in reference (3).

We investigated the thixotropic behavior of paints modified with different HEURs using the 3ITT, which is a key method for assessing the time-dependent behavior of paints, simulating conditions from rest to application^3,67−70^ (Figure 11a). This test comprises three intervals simulating a paint’s condition at rest, during high shear applications such as brushing, and the subsequent rest phase. The thixotropic index (TI) was calculated to quantify this behavior, and the method for this calculation can be found in the Supporting Information.

Our findings reveal that the choice of HEUR significantly affects a paint’s viscosity recovery rates and thus its TI values (Figure 11b). For instance, the paint modified with the most hydrophobic HEUR (paint 7 in Figure 11) and the most pseudoplastic behavior exhibited rapid viscosity recovery and the highest TI value, whereas paints 2, 5, and 6 with the weaker hydrophobic part and the most Newtonian behavior with the lowest viscosities exhibited the lowest TI values and were slower to recover viscosity. Additionally, molecular weight plays a crucial role; doubling the molecular weight of HEURs from approximately 15,000 to 30,000 g/mol led to an increase in the TI by over 8 orders of magnitude, as exemplified by the comparison between paint 8 modified with a HEUR molecular weight of 14,000 g/mol and paint 4 with a molecular weight of 33,000 g/mol.

Practical applications of our findings highlight the crucial role of end-use performance in paint formulations, particularly by focusing on leveling and sagging properties as key quality indicators. Using standard Leneta charts for evaluation, we found a clear correlation between the molecular structure of the HEUR and paint performance. Paint 7 that has the most hydrophobic segment and paint 4 that has the highest molecular weight showed superior antisagging properties but lacked in leveling rating due to their high TI and quick viscosity recovery. On the other hand, paints 2, 5, and 6 having weaker hydrophobic segments displayed better leveling performance but showed poor sag resistance due to their low TI values. Paints with molecular weights between 14,000 and 23,000 g/mol demonstrated a more balanced performance in both leveling and sagging. These insights are critical as real-world paints often contain more than one HEUR thickener of different chemical structure to achieve optimal performance on both leveling and sagging rating.

#### 3.4.4 Thermal Stability Measurements

**Figure 12:**
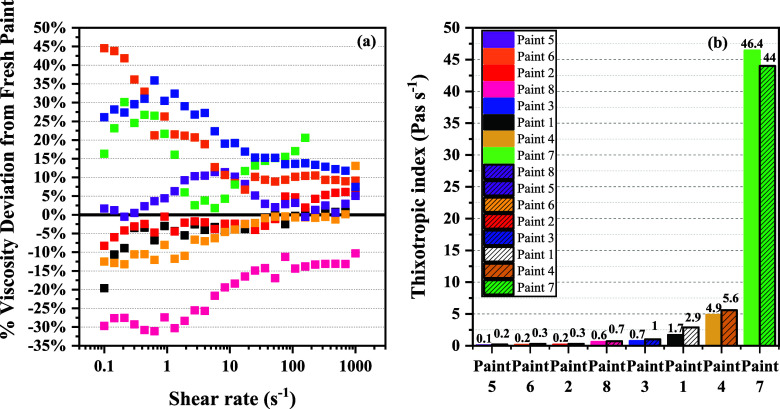
(a) Percent difference in shear viscosity across the entire shear rate range for fresh and aged paints (positive values indicate higher viscosities of the aged samples compared to the fresh paints). (b) Thixotropic index for fresh and aged paints.

In an accelerated aging test for paint samples, we assessed the storage stability and the effects of different HEUR thickeners. Figure 12 displays viscosity changes and thixotropic indices for both the fresh and aged samples. Notably, no phase separation was observed for any of the aged paints. Figure 12a highlights that aging primarily influences viscosity in low shear regions. Paints 1, 4, 2, and 5 showed a low viscosity deviation (±15%) compared to their fresh counterparts with no clear correlation to HEUR structures. Further, a general trend of increased TI values is observed as HEUR molecular weight increases (Figure 11b). The absence of any clear correlation of viscosity change in the aged paints, compared to their fresh counterparts, with the chemical structure of the HEUR thickener, based on the results of Figure 12, is not surprising. It can be attributed to the fact that the effect of accelerated paint aging on paint viscosity is paint-specific; that is, it may promote or disrupt interparticle associations, which in turn affects the rheological properties. The enhancement of rheological properties could also result from thermal degradation reactions within the polymer–binder–latex matrix. Such reactions might cause a molecular recombination, ultimately giving rise to a more robust and chemically stabilized network with an enhanced thermal stability. Similar observations regarding thermoplastic polyurethane systems have been made by other authors.^71^ For a more detailed analysis of the results of steady shear and oscillatory tests between fresh and aged paints, please refer to the Supporting Information.

## 4 Conclusions

This work combines experimental and computational approaches to provide a comprehensive evaluation of the impact of the HEUR chemical structure on aqueous solutions, latex-based emulsions, and waterborne paints. Linear HEUR thickeners with different hydrophilic and hydrophobic segments were synthesized through a controlled one-step polymerization process by employing PEGs with molecular weights ranging from 2000 to 10,000 g/mol, resulting in HEUR molecular weights ranging from 8000 to 33,000 g/mol. To investigate variations in the molecular weight, we used HMDI-C8 as the terminal hydrophobic group. To investigate the effect of the hydrophobic segment, we selected a HEUR molecular weight of 23 000 g/mol. Utilizing diisocyanates such as HMDI, IPDI, and HDI, we standardized the end-capping with C8. When monoalcohol lengths were altered (C6 to C12), HMDI was retained as the diisocyanate linker.

The rheological analysis demonstrated a significant influence of the hydrophobe’s structure on HEUR behavior across all studied formulations. Notably, HEUR samples with increased terminal hydrophobicity, particularly P8-HMDI-C12, exhibited a strongly pseudoplastic behavior in all formulations studied. In paint formulations, these structures demonstrated rapid structural regeneration and high TI values, resulting in enhanced sag resistance. However, these benefits were offset by compromised leveling properties. In contrast, HEURs with less effective hydrophobic segments (P8-HDI/IPDI-C8 and P8-HMDI-C5/C6) displayed a Newtonian rheological behavior, and the corresponding paint formulations showed slower structural regeneration with superior leveling but worse antisag performance.

Regarding the hydrophilic segment, the gradual increase in HEUR molecular weight up to 23,000 g/mol resulted in marginal viscosity changes in aqueous solutions, whereas a pronounced viscosity increase was observed with a molecular weight of 33,000 g/mol. In latex emulsions, lower-molecular-weight HEURs (8000 g/mol) displayed extended flocculated regions, a trend that appeared to be diminished with a 14,000 g/mol HEUR and absent with a 23,000 g/mol HEUR. In paint formulations, molecular weights of 14,000, 18,000, and 23,000 g/mol exhibited similar rheological response in paint formulations, but a molecular weight of 33,000 g/mol deviated, showing a shift toward higher viscosities and solid-like properties.

Finally, to explore HEUR micellar morphology, we employed coarse-grained molecular dynamics (CG-MD) simulations that effectively captured the spontaneous micelle formation starting from random polymeric chain distributions. Although the CG-MD model aligned well with experimental observations, we acknowledge its limitations, especially in capturing hydrophobic interactions for very long polymer backbones (structure of diisocynate linker and hydrophilic backbone). Future studies could further explore these limitations and potentially refine the modeling approach (e.g., by developing different force fields). We introduced an innovative automated approach for micellar identification using the DBSCAN algorithm, accurately computing distinct hydrophobic micelle cores. By performing CG-MD simulations for various concentration values in aqueous solutions, we observed variations in the micellar network density correlating with experimental viscosity trends, whereas as the hydrophilic length of HEUR increased, the micellar volume continued to grow in alignment with observed experimental viscosity changes.

## Supporting Information Available

More details on the aqueous HEUR solutions, MD simulations, latex-based emulsions, and paint formulations (PDF)
