# Supplementary material for: Tailoring Waterborne Coating Rheology with Hydrophobically Modified Ethoxylated Urethanes (HEURs): Molecular Architecture Insights Supported by CG-MD Simulations
Source: Ind Eng Chem Res. 2024 May 24;63(22):10009–26. doi: 10.1021/acs.iecr.4c00253 (PMC11190988; doi:10.1021/acs.iecr.4c00253)
Supplement: Supplementary file 1 — ie4c00253_si_001.pdf [file ie4c00253_si_001.pdf]

# Supporting Information

## Tailoring Waterborne Coatings Rheology with Hydrophobically Modified Ethoxylated Urethanes (HEURs): Molecular Architecture Insights Supported by CG-MD Simulations

**Ioanna Tzortzi[a], Imane Joundi[c], Michail Kavousanakis[a],Theodora Spyriouni[d], Ariana Bampouli[b], Guillaume Michaud[c], Tom Van Gerven[b], Georgios D. Stefanidis[a*]**

[a] *School of Chemical Engineering National Technical University of Athens, Iroon Polytecneiou 9, Zografou Campus, 157 80, Athens, Greece.*

[b]*Department of Chemical Engineering, KU Leuven, Celestijnenlaan 200F, B-3001 Leuven, Belgium.*

[c] *COATEX SAS, 69730 Genay, France*

[d] *SCIENOMICS SAS, 16 Rue de l'Arcade 75008 Paris, France*

[*]*Corresponding author's email: gstefani@mail.ntua.gr*

# 1. Aqueous solutions

## Coarse-Grained MD simulations

**Table S1.** MARTINI force-field parameters.

| Bead types and mass | PEO: 44.05 g/mol<br>P3 (Polar – Degree of polarity 3): 57.05 g/mol<br>SC1CH (Cyclohexane bead): 28 g/mol<br>C1 (Apolar – Degree of polarity 1): 58 g/mol<br>P4 (Polar – Degree of polarity 4): 72 g/mol | | |
|---|---|---|---|
| Non-bonded Lennard Jones interactions | $U_{ij}(r) = 4\varepsilon_{ij}\left[\left(\frac{\sigma_{ij}}{r}\right)^{12} - \left(\frac{\sigma_{ij}}{r}\right)^{6}\right]$ | | |
| | $i-j$ | $\sigma_{ij}$ (nm) | $\varepsilon_{ij}$ (kJ mol$^{-1}$) |
| | P4-P4 | 0.47 | 5 |
| | P4-PEO | 0.47 | 4 |
| | P4-C1 | 0.47 | 2 |
| | P4-P3 | 0.47 | 5 |
| | P4-SC1CH | 0.47 | 2 |
| | PEO-PEO | 0.43 | 3.375 |
| | PEO-P3 | 0.47 | 4.5 |
| | PEO-SC1CH | 0.43 | 2.026 |
| | PEO-C1 | 0.47 | 2.7 |
| | P3-P3 | 0.47 | 5 |
| | P3-SC1CH | 0.47 | 2.3 |
| | P3-C1 | 0.47 | 2.3 |
| | SC1CH-SC1CH | 0.43 | 2.625 |
| | SC1CH-C1 | 0.47 | 3.5 |
| | C1-C1 | 0.47 | 3.5 |
| Bonded Interactions | $V_b(r) = \frac{1}{2}k_{ij}^{b}\left(r - b_{ij}\right)^2$ | | |
| | Bond | $k_{ij}^{b}$ (kJ mol$^{-1}$nm$^{-2}$) | $b_{ij}$ (nm) |
| | PEO-PEO | 17000 | 0.33 |
| | PEO-P3 | 1250 | 0.47 |
| | P3-SC1CH | 1250 | 0.47 |
| | P3-C1 | 1250 | 0.47 |
| | C1-C1 | 1250 | 0.47 |
| | SC1CH-SC1CH | 5000 | 0.3 |

| Bond angle bending | $V_a(\theta) = \frac{1}{2} k^a_{ijk} \left(\cos(\theta) - \cos(\theta_{ijk})\right)^2$ | | |
|---|---|---|---|
| | Bond angle | $k^a_{ijk}$ (kJ mol$^{-1}$rad$^{-2}$) | $\theta_{ijk}$ (deg) |
| | PEO-PEO-P3 | 25 | 179.9 |
| | PEO-PEO-PEO | 85 | 130 |
| | SC1CH-SC1CH-SC1CH | 25 | 179.9 |
| | PEO-P3-SC1CH | 25 | 179.9 |
| | SC1CH-SC1CH-P3 | 25 | 179.9 |
| | P3-C1-C1 | 25 | 179.9 |
| | C1-P3-SC1CH | 25 | 179.9 |
| | P3-SC1CH-SC1CH | 25 | 179.9 |
| | SC1CH-P3-C1 | 25 | 179.9 |

## 2. Latex-formulations

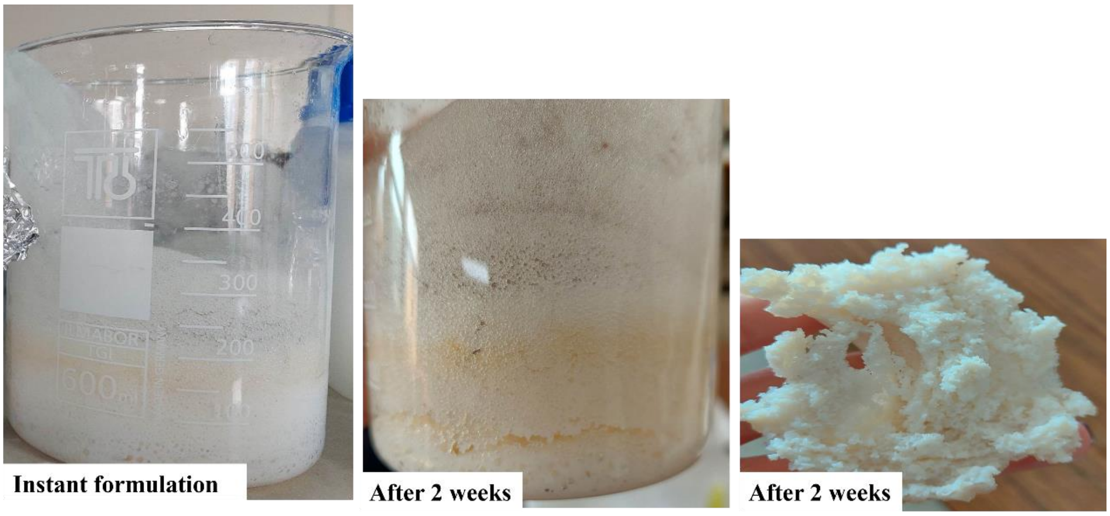


**Figure S1.** Picture of the instant phase separation of HEUR: P2-HMDI-C8, Mn=8,000 g/mol upon formulation with latex.

# 3. Paint formulations

**Table S2.** Indices of Pseudoplasticity, Yield Points, G' Values (derived from the linear plateau of the AS test), and Crossover Points (observed in the FS test) for all paints, arranged in ascending order.

| | **Pseudoplasticity Index** $\left(\frac{\eta_{0.1\,s^{-1}}}{\eta_{10^4\,s^{-1}}}\right)$ | | **Yield point (γ%)** | | **G’ (average value calculated within the LVER) (Pa)** | | **Crossover Point (Hz)** |
|---|---|---|---|---|---|---|---|
| **Paint 2** | 23 | **Paint 5** | 3,15 | **Paint 5** | 6 | **Paint 8** | - |
| **Paint 5** | 34 | **Paint 1** | 4,63 | **Paint 2** | 13 | **Paint 3** | 0,46 |
| **Paint 6** | 36 | **Paint 2** | 4,63 | **Paint 6** | 15 | **Paint 1** | 0,68 |
| **Paint 3** | 53 | **Paint 6** | 4,63 | **Paint 3** | 30 | **Paint 2** | 1,47 |
| **Paint 8** | 62 | **Paint 8** | 6,80 | **Paint 8** | 33 | **Paint 7** | 1,47 |
| **Paint 1** | 75 | **Paint 3** | 6,80 | **Paint 1** | 36 | **Paint 6** | 1,72 |
| **Paint 4** | 139 | **Paint 4** | 6,81 | **Paint 4** | 63 | **Paint 5** | 3,16 |
| **Paint 7** | 658 | **Paint 7** | 14,73 | **Paint 7** | 113 | **Paint 4** | 3,69 |

## Oscillatory measurements

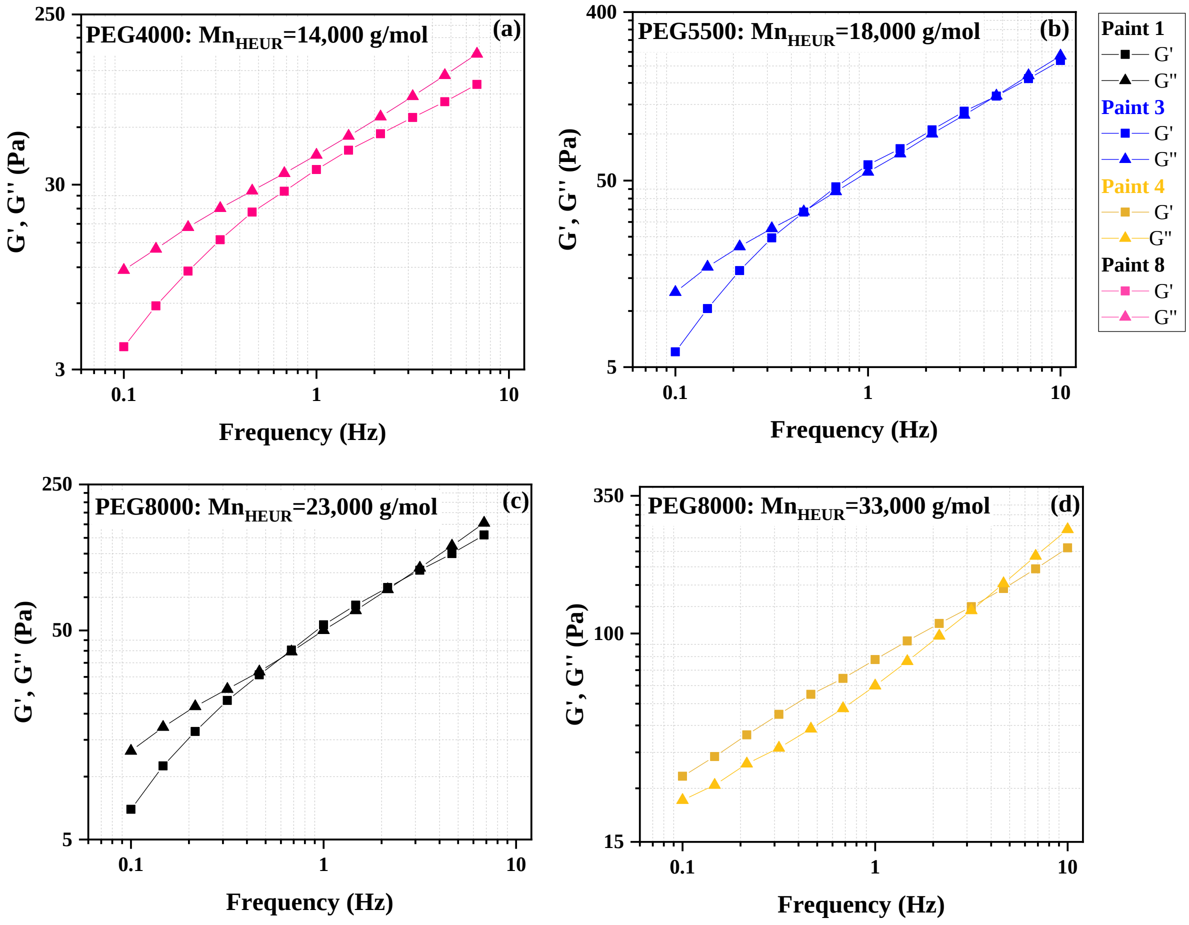


**Figure S2**. Frequency sweep tests for paints thickened with HEURs with varying hydrophilic segment (a) 14,000 g/mol (b) 18,000 (c) 23,000 g/mol (d) 33,000 g/mol.

## Calculation of Thixotropic Index

The 3ITT test comprises three key intervals:

**Interval (1):** A very low shear rate is applied to simulate the paint's behavior at rest.

**Interval (2):** A high shear rate is applied to simulate the structural breakdown of the paint during application processes such as brushing or rolling.

**Interval (3):** A very low shear rate is reapplied to simulate the paint's structural regeneration at rest.

The TI has previously been defined as:[64]

$$TI = \frac{\eta_{t_R} - \eta_{HS}}{t_R}$$

$\eta_{t_R}$: was selected to be 75% of the low shear viscosity.

$\eta_{HS}$: viscosity at the end of the high shear interval (2nd interval)

$t_R$: recovery time to regain $\eta_{t_R}$

## Thermal Stability of Paints

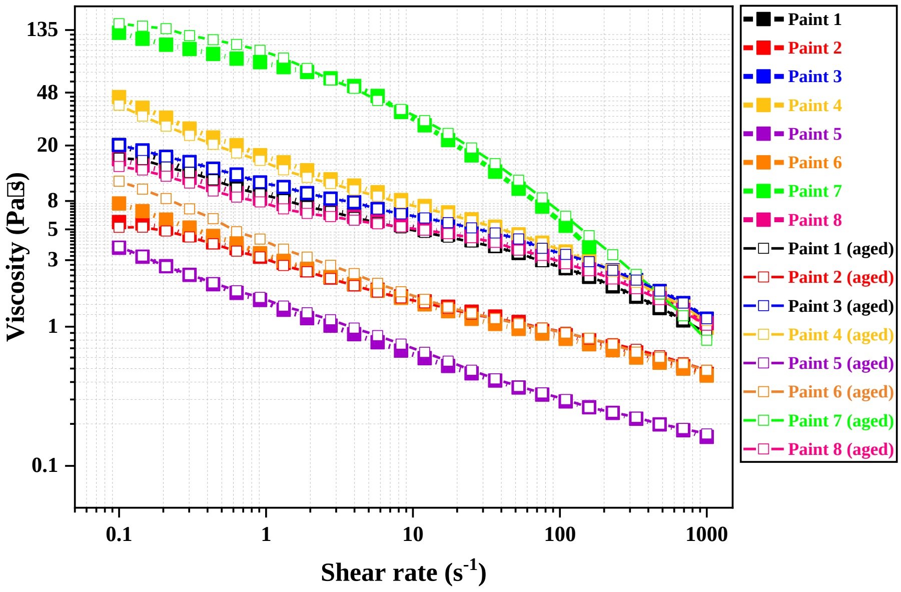


**Figure S3**. Steady shear viscosity curves for fresh and aged Paints.

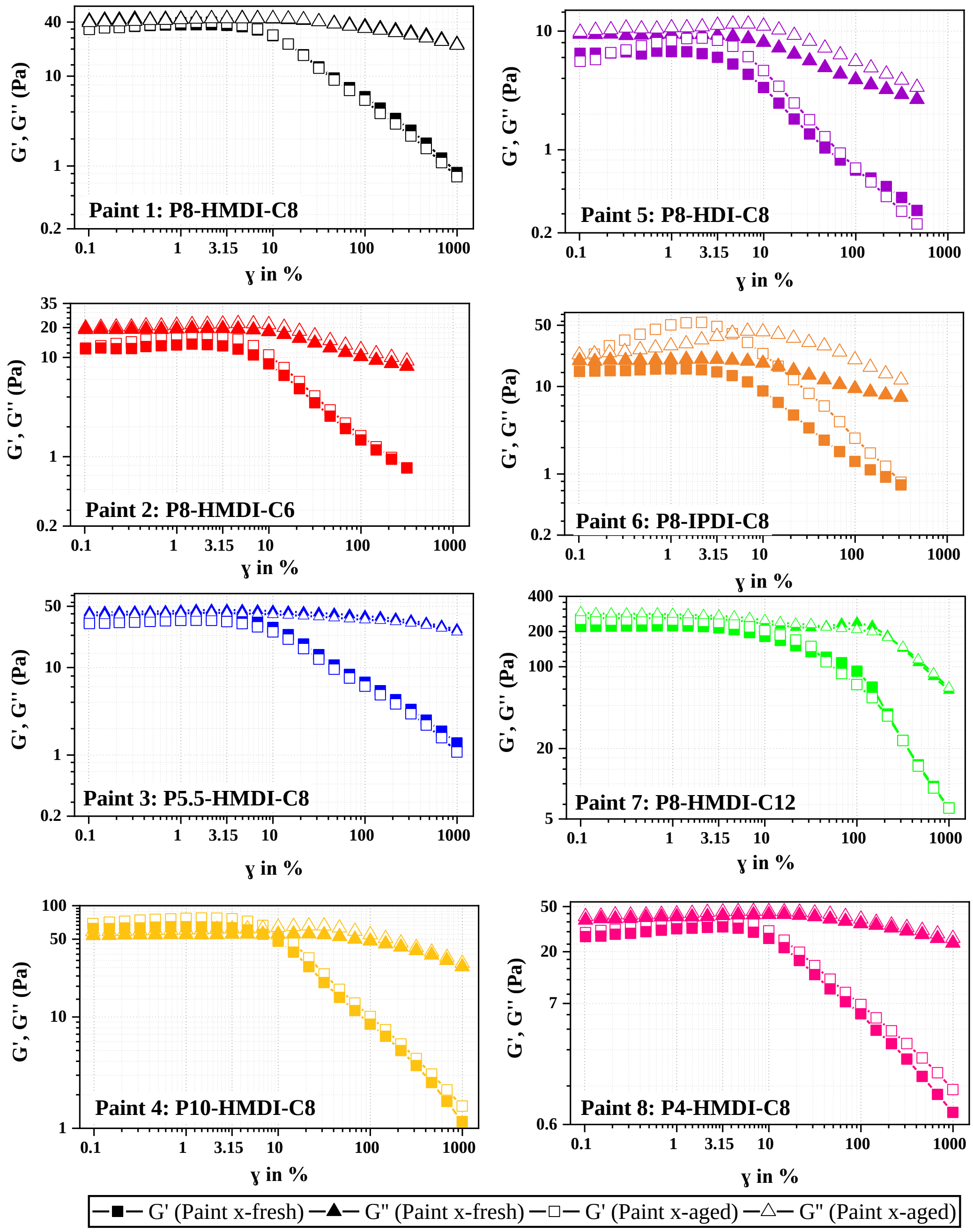


**Figure S4.** AS test for fresh and aged Paints.

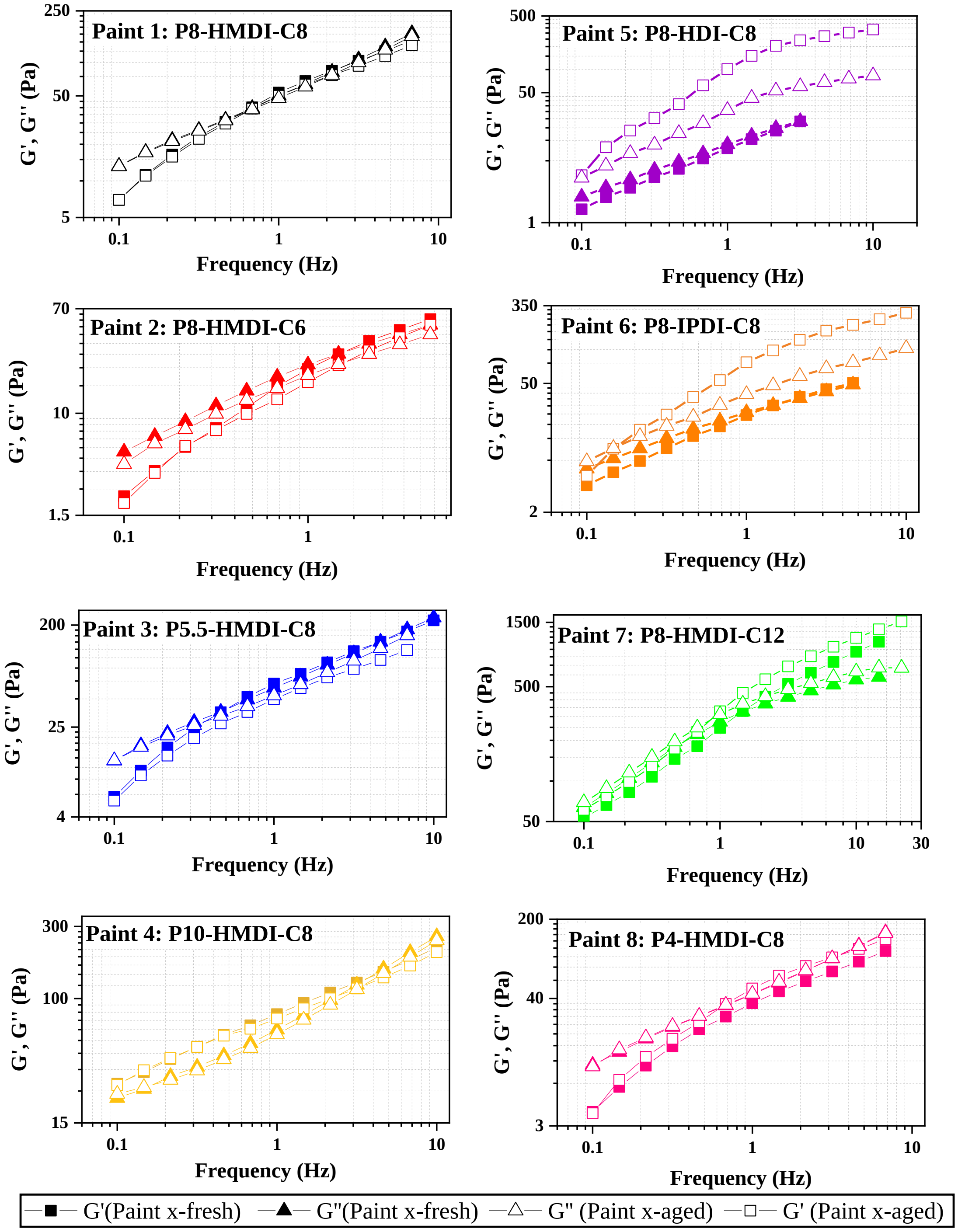


**Figure S5.** FS test for fresh and aged Paints.

Table S4 provides a summary of observations derived from the comparisons of the AS and FS tests between the fresh and aged samples. AS and FS test are presented in Figures S3 and S4.

The AS results didn't show significant variations; however, the most distinct rheological behavior, indicative of superior structural strength, was evident in Paints 2, 5, and 6. The FS results bolstered these findings. Most notably, Paint 5 and Paint 6 presented superior viscoelastic properties. Both had a crossover point at an exceedingly low frequency, showcasing predominantly solid behavior at the rest of the frequency domain. This solid character implies robust interparticle associations, with superior properties compared to the original network observed in the fresh paints. Overall, taking into consideration the results of all the rheological tests, Paint 1 displayed the most stable behavior, while Paint 4 had the least deviation compared to its fresh counterparts.

**Table S3.** Summary of observations derived from the comparisons of the AS and FS tests between the fresh and aged samples.

| | **Amplitude Sweep** | **Frequency Sweep** |
|---|---|---|
| Paint 1 | The values of G' and G'' remain consistent and exhibit similar trends throughout the entire % strain range. | The values of G' and G'' remain consistent and exhibit similar trends throughout the entire frequency range. Their crossover points are also identical. |
| Paint 2 | Nearly identical G' and G'' curves were observed, with G' exhibiting an upward trend in the LVER, indicating a thickening effect. | The crossover is observed at the same frequency for both G' and G''(f=1.46 Hz). While the trends of both modulus remain consistent, the aged paint exhibits diminished values. |
| Paint 3 | Nearly identical G' and G'' curves were observed, with a slight tendency towards decreased values. | Compared to the fresh paint, the aged paint lacks a crossover point. Throughout the entire frequency range, the aged paint distinctly exhibits a viscous behavior with G'' consistently greater than G'. |

| | | |
|---|---|---|
| Paint 4 | Nearly identical G' and G" curves were observed, with a slight tendency towards increased values. | Both the fresh and aged paints exhibit the same crossover point. While the G' and G" curves follow a similar trend for both modulus, the aged paint displays marginally reduced values. |
| Paint 5 | Nearly identical G' and G" curves were observed, with G' exhibiting an upward trend in the LVER, indicating a thickening effect. | There's a marked effect on the G', G" curves. The aged samples exhibit a crossover point at very low frequencies, showcasing a predominantly solid behavior throughout most of the frequency range. Both modulus also tend to reach a plateau value. In contrast, the fresh sample has its crossover point at a higher frequency, displaying a liquid-like behavior in the low-frequency region. At the higher frequency domain, the fresh sample presents equivalent values for both G" and G'. |
| Paint 6 | A shift from a liquid-like behavior (G' < G") to a solid-like characteristic was noted, accompanied by a thickening effect in both G' and G" at lower strain values. | Paint 6 shows approximately the same tendency as Paint 5. |
| Paint 7 | Nearly identical G' and G" curves were observed | The crossover occurs at a lower frequency with elevated G' and G" values, though the curves largely maintain a similar trend. There's a more pronounced deviation between the G' and G" curves in the high-frequency domain. |
| Paint 8 | Nearly identical G' and G" curves were observed, with a slight tendency towards increased values. | In contrast to the fresh sample, which doesn't exhibit a crossover, the aged sample displays a crossover point at f=0.68 Hz and primarily demonstrates a liquid behavior at low frequencies. At higher frequencies, the G' and G" values converge and become closely aligned. |
